# The mutational profile in a South African cohort with inherited neuropathies and spastic paraplegia

**DOI:** 10.3389/fneur.2023.1239725

**Published:** 2023-08-29

**Authors:** Amokelani C. Mahungu, Elizabeth Steyn, Niki Floudiotis, Lindsay A. Wilson, Jana Vandrovcova, Mary M. Reilly, Christopher J. Record, Michael Benatar, Gang Wu, Sharika Raga, Jo M. Wilmshurst, Kireshnee Naidu, Michael Hanna, Melissa Nel, Jeannine M. Heckmann

**Affiliations:** ^1^Neurology Research Group, Division of Neurology, Department of Medicine, University of Cape Town, Cape Town, South Africa; ^2^Neuroscience Institute, University of Cape Town, Cape Town, South Africa; ^3^Department of Neuromuscular Diseases, Queen Square Institute of Neurology, University College London, London, United Kingdom; ^4^Department of Neuromuscular Disease, Queen Square UCL Institute of Neurology and the National Hospital of Neurology and Neurosurgery, London, United Kingdom; ^5^Department of Neurology, University of Miami Miller School of Medicine, Miami, FL, United States; ^6^Center for Applied Bioinformatics, St. Jude Children's Research Hospital, Memphis, TN, United States; ^7^Division of Paediatric Neurology, Department of Paediatrics and Child Health, Red Cross War Memorial Children's Hospital, University of Cape Town, Cape Town, South Africa; ^8^NHS Highly Specialised Service for Rare Mitochondrial Disorders, Queen Square Centre for Neuromuscular Diseases, The National Hospital for Neurology and Neurosurgery, London, United Kingdom

**Keywords:** whole exome sequencing, whole genome sequencing, Charcot-Marie-Tooth disease, hereditary spastic paraplegia, African, equity, diversity and inclusion

## Abstract

**Introduction:**

Limited diagnostics are available for inherited neuromuscular diseases (NMD) in South Africa and (excluding muscle disease) are mainly aimed at the most frequent genes underlying genetic neuropathy (GN) and spastic ataxias in Europeans. In this study, we used next-generation sequencing to screen 61 probands with GN, hereditary spastic paraplegia (HSP), and spastic ataxias for a genetic diagnosis.

**Methods:**

After identifying four GN probands with *PMP22* duplication and one spastic ataxia proband with SCA1, the remaining probands underwent whole exome (*n* = 26) or genome sequencing (*n* = 30). The curation of coding/splice region variants using gene panels was guided by allele frequencies from internal African-ancestry control genomes (*n* = 537) and the Clinical Genome Resource's Sequence Variant Interpretation guidelines.

**Results:**

Of 32 GN probands, 50% had African-genetic ancestry, and 44% were solved: *PMP22* (*n* = 4); *MFN2* (*n* = 3); one each of *MORC2, ATP1A1, ADPRHL2, GJB1, GAN, MPZ*, and *ATM*. Of 29 HSP probands (six with predominant ataxia), 66% had African-genetic ancestry, and 48% were solved: *SPG11* (*n* = 3); *KIF1A* (*n* = 2); and one each of *SPAST, ATL1, SPG7, PCYT2, PSEN1, ATXN1, ALDH18A1, CYP7B1*, and *RFT1*. Structural variants in *SPAST, SPG11, SPG7, MFN2, MPZ, KIF5A*, and *GJB1* were excluded by computational prediction and manual visualisation.

**Discussion:**

In this preliminary cohort screening panel of disease genes using WES/WGS data, we solved ~50% of cases, which is similar to diagnostic yields reported for global cohorts. However, the mutational profile among South Africans with GN and HSP differs substantially from that in the Global North.

## 1. Introduction

Little is known about the underlying genetic causes of neuromuscular disorders (NMD) among African subpopulations, especially among those from sub-Saharan Africa (1, 2). This may be due to several factors, the most important of which is limited resources. In a recent review of genetic evidence related to the causes of genetic neuropathies (GN), including hereditary motor sensory neuropathies [hereafter referred to as Charcot-Marie-Tooth disease (CMT)], hereditary spastic paraplegias (HSP), and spinal muscular atrophy (SMA) in Africans, we found reports of disease variants from only nine HSP and three CMT probands from six sub-Saharan African countries (Ivory Coast, Kenya, Mali, Nigeria, Somalia, and South Africa) (2). Six of these nine reports used next-generation sequencing to screen disease genes, while three performed single-gene testing.

In South Africa, limited genetic testing is available for non-muscle NMD and includes only *PMP22* multiplex ligation-dependent probe amplification (MLPA) for GN and spinocerebellar ataxias 1, 2, 3, 6, 7, 12, 17 and Frataxin expansion screening for spastic ataxias. To address this unmet need, we have leveraged resources from two international consortia to drive the first genetic analysis of a South African cohort with inherited NMD using next-generation sequencing and virtual gene panel analysis. Here, we describe the genetic findings in patients presenting with genetic neuropathies, hereditary spastic paraplegias, and related disorders such as spastic ataxias.

## 2. Methods

### 2.1. NMD probands

Patients were eligible if they had a long history of a phenotype resembling a neuromuscular genetic disorder, either with a primary component of neuropathy or spastic paraplegia, or as part of a complex disorder with ataxia, and with or without a family history (3). The study cohort consisted of 61 probands, which were categorised using their main presenting clinical features as genetic neuropathies (GN) or hereditary spastic paraplegia (HSP), the latter group including those with associated ataxia and those with ataxia as the predominant clinical feature (designated spastic ataxia).

Almost all study participants were phenotyped and recruited opportunistically at Groote Schuur Hospital, University of Cape Town, South Africa (SA), between 2017 and 2022, whereas two HSP probands underwent prior phenotyping, and archived DNA was used. One HSP proband was assessed at the Red Cross Children's Hospital (SR, JMW).

The study was approved by the University of Cape Town Human Research Ethics Committee, and all patients gave informed consent to participate, including the sharing of case details and images.

#### 2.1.1. Genetic testing strategy

Thirty probands were recruited for the International Centre for Genomic Medicine in Neuromuscular Diseases (ICGNMD) consortium and underwent whole exome sequencing (WES), and five HSP probands underwent whole genome sequencing (WGS) as part of the Clinical Research in ALS and Related Disorders for Therapeutic Development (CReATe) Consortium Phenotype Genotype Biomarker study (NCT02327845). An additional 25 probands underwent WGS at service providers in Cape Town, including the resequencing of five probands who had ICGNMD WES (Supplementary Table 1). One proband had an NMD gene panel test performed by Invitae.

Study participants with either demyelinating or intermediate conduction velocities (CVs) on electrophysiological testing underwent the MLPA (Salsa MLPA kit P405 CMT1, MRC-Holland, Amsterdam) for *PMP22* copy number analysis by the SA National Health Laboratory Services (NHLS) prior to WES. Those with spastic ataxia had undergone repeat expansion testing for spinocerebellar ataxia (SCA) types 1, 2, 3, 6, 7, 12, 17 and Frataxin (NHLS). No further testing for large deletions or duplications was performed through MLPA analysis.

### 2.2. Control datasets

Whole genome sequencing data from various projects were aggregated to generate an internal African-ancestry database for allele frequency filtering (*n* = 537): 100 individuals from the AWI-Gen study (4) and 347 individuals from the H3Africa Genotyping Chip project (5), 39 individuals from the Simons Genome Diversity project (6), 24 individuals from the South African Human Genome program (7), and 27 individuals from other studies (1, 8). DNA was sequenced by various service providers using both PCR and PCR-free sequencing kits, where 100–150 bp read-length sequencing libraries were obtained and sequenced to a coverage of 30× (Supplementary Table 1).

### 2.3. Next-generation sequencing

DNA from whole blood was extracted for next-generation sequencing (NGS) using previously described methods (9). WES or WGS on NMD probands was performed using various library kits and sequencing platforms (Supplementary Table 1).

### 2.4. Alignment and variant calling

The NGS data were analysed at UCT using the *Ilifu* Cloud Computing Facility. WES and WGS reads were aligned to the NCBI GRCh38 reference genome with ALT contigs using alt-aware alignment, followed by joint variant calling according to the Genome Analysis Toolkit (GATK) best-practice guidelines for exomes and genomes (documented in https://github.com/grbot/varcall), which was performed separately for WES and WGS datasets.

### 2.5. NMD gene variant annotation and filtering

Both the case and control joint called VCF files were uploaded to the NHGRI AnVIL (10) into various workspaces, which were then loaded into seqr, a rare disease analysis platform developed by the BROAD Institute (11). As part of the seqr's loading pipeline, multiallelic sites were split and then annotated with VEP version 95. High and moderate impact variants (missense, nonsense, essential and extended splice site, frameshift, and in frame) were assessed for both *de novo*/dominant and recessive inheritance patterns using gnomAD minor allele frequency (MAF) thresholds of 0.001 and 0.01, respectively. Further curation was restricted to variants occurring in genes represented in the following PanelApp Australia gene panels (12): hereditary neuropathy CMT isolated and complex (version 1.63), optic atrophy (version 1.17), hereditary spastic paraplegia superpanel (version 2.64), and ataxia superpanel (version 3.6). Panel selection was guided by clinical phenotype, although probands were deemed unsolved only when the genes from all four panels screened negative for candidate variants.

### 2.6. Variant curation and classification according to ACMG guidelines

In the absence of ClinGen CMT and HSP variant curation expert panel guidelines, candidate variants were curated in the ClinGen Variant Curation Interface (VCI) (13) according to the American College of Medical Genetics (ACMG) guidelines (14) with the modifications recommended by the ClinGen Sequence Variant Interpretation working group (15) (detailed in Supplementary Table 2). In addition to the gnomAD database, the rarity of variants was confirmed by assessing their frequency in an internal dataset of African-ancestry control genomes [*n* = 537, 151 (28%) from South Africa; see Methods 2.2]. We used the Mastermind search engine (16) to perform comprehensive literature searches for candidate variants and contacted various genetic testing laboratories for additional information regarding their ClinVar variant submissions (e.g., phenotype information and internal laboratory variant frequency information), which proved helpful in classifying variants. The NGS read support for each variant was verified (read depth, genotype quality, and allelic balance), and selected variants were validated by Sanger sequencing. The interpretations for each classified variant (pathogenic, likely pathogenic, or uncertain significance) have been submitted to ClinVar (https://www.ncbi.nlm.nih.gov/clinvar/).

### 2.7. Analysis of structural variants

In unsolved cases (those without short disease-causing variants such as single nucleotide variants and small insertions/deletions in the various gene panels), the following genes were screened for structural variants using a combination of computational prediction by ClinSV (17) and manual visualisation using the Integrative Genomics Viewer (IGV) (18) and Samplot (19): *SPAST, SPG11, SPG7, MFN2, MPZ, KIF5A*, and *GJB1*. To control for batch effects related to the library preparation and sequencing instruments, exomes and genomes from the same sequencing batch were compared to identify split reads, discordant pairs, and coverage anomalies indicative of structural variation.

### 2.8. Statistical analysis

We utilised GraphPad Prism v9.5.1 to perform statistical analysis on the probands' ages of onset. We assessed the continuous variable (ages of onset) using the nonparametric Mann–Whitney *U*-test for skewed data by comparing the ages of onset between solved and unsolved cases as median values with their interquartile ranges (IQR).

## 3. Results

### 3.1. Patient demographics

Probands self-categorised themselves according to South Africa census population categories as South African Coloured (SAC, *n* = 25), Black African (*n* = 10), and European-genetic ancestry or White (*n* = 25). Furthermore, our cohort included one proband with Indian-genetic ancestry. The majority (57%) of our study cohort was of African-genetic ancestry (*n* = 35, combined B and SAC; Table 1). This information is relevant for genomic analysis as the African ancestry probands in our cohort have no ancestry-matched population data represented in the gnomAD database, which impacts the effectiveness of variant frequency filtering to identify rare variants in these individuals. The SAC ancestry group is an admixed population primarily residing in the Western Cape Province of South Africa whose genetic ancestry is mainly from Khoisan (hunter-gatherers) and Black African populations (60%) with fewer contributions from South-East Asians (20%) and Europeans (20%).

**Table 1:** Characteristics of South African patients with genetic neuropathies (GN), hereditary spastic paraplegia (HSP), and spastic ataxia.

	**GN (*****n*** = **32)**			**HSP and spastic ataxia (*****n*** = **29)**		**Combined (*n* = 61)**
	**CMT (*****n*** = **21)**	**HMN (*****n*** = **7)**	**Other (*****n*** = **4)**	**HSP (*****n*** = **23)**	Spastic ataxia^*^(***n*** = **6)**	
Median AAO^#^ (IQR)	13 (9–19)	17 (13–25)	12 (2–45)	15 (9–24)	15 (6–25)	15 (9–20)
Male sex, *n* (%)	9 (43)	5 (71)	2 (50)	6 (22)	4 (67)	26 (43)
**Genetic ancestry**						
African *n* (%)	10 (48)	5 (71)	1 (25)	15 (61)	4 (67)	35 (57)
European *n* (%)	11 (52)	2 (29)	3 (75)	8 (35)	1 (17)	25 (42)
Indian *n* (%)	0	0	0	0	1 (17)	1 (1)
Solved *n* (%)	11 (52)	2 (29)	1 (25)	12 (52)	2 (33)	28 (46)

GN, genetic neuropathies; HSP, hereditary spastic paraplegia; CMT, Charcot-Marie-Tooth disease; HMN, hereditary motor neuropathy (based on electrophysiological evidence of normal sensory nerve action potentials); AAO, age at onset (^#^years); IQR, interquartile range between 25th and 75th centile.

* Refers to probands with ataxia as the predominant clinical feature. Solved refers to probands in whom a pathogenic or likely pathogenic variant was identified.

The cohort comprised 61 individuals, of whom 32 were broadly categorised as GN and 29 as HSP, although in six of the 29 probands, ataxia was the predominant clinical feature. Among those with GN, most had CMT (21/32, 66%) with electrophysiological evidence of both sensory and motor impairment, and 7/32 (22%) had hereditary motor neuropathy (HMN) with normal sensory nerve action potentials (SNAPs) on electrophysiological testing (Table 1). Only four individuals had a demyelinating CMT1 phenotype with motor conduction velocities <38 m/s, and one individual had severe hypertrophic nerves and was previously recorded as having a “demyelinating picture”; she was too disabled at recruitment for electrophysiological testing. One individual had ataxia, neuropathy, and cognitive impairment without telangiectasia.

HSP categorisation referred to a predominant spastic phenotype involving the legs more than arms (*n* = 8), which may be complicated by other neurological signs (e.g., ataxia, neuropathy; *n* = 15), whereas six probands were classified as having spastic ataxia if the clinical picture was predominantly ataxia with additional spasticity (Table 1).

### 3.2. Genetic findings

**Figure 1:**
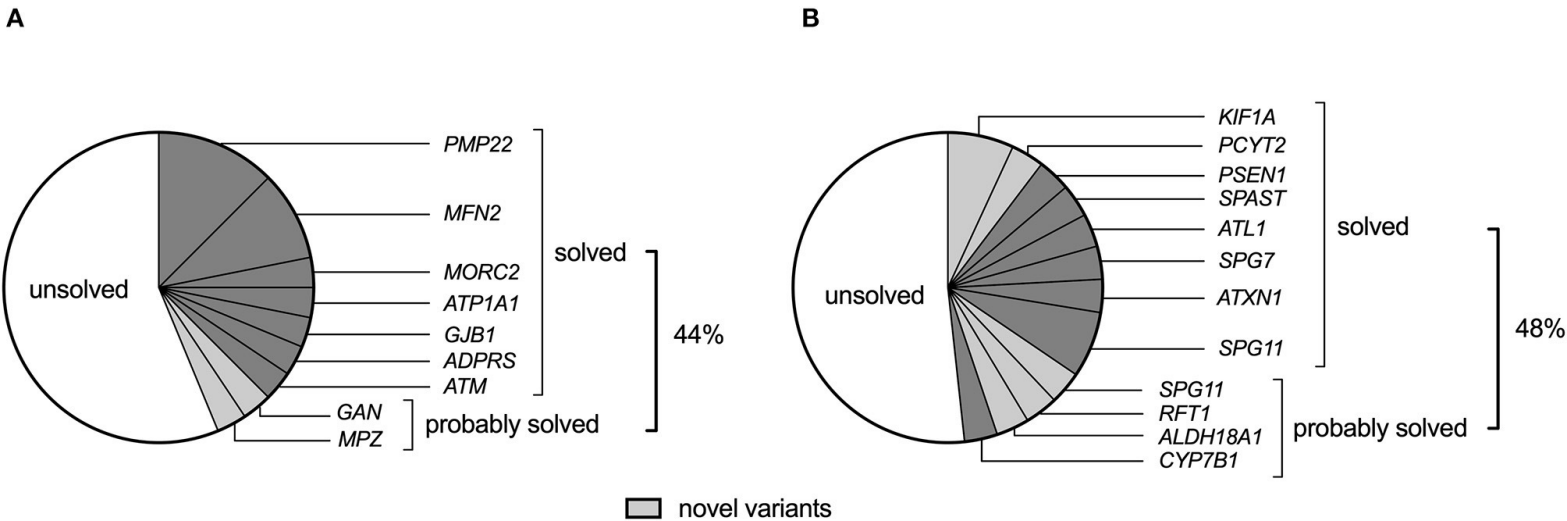
The genetic landscape of **(A)** genetic neuropathy (GN) and **(B)** hereditary spastic paraplegia (HSP) (including spastic ataxias) in a South African cohort of 61 probands. Novel variants (light grey) refer to variants that have not been previously associated with the disease.

Apart from detecting four *PMP22* gene duplications in GN (CMT1) and 1 *ATXN1* expansion mutation among HSP probands, the remaining probands underwent WES and/or WGS for gene panel analysis. With the rigorous and objective application of ACMG evidence codes (described in methods 2.6 and Supplementary Table 2), we identified 10 pathogenic, eight likely pathogenic, and 11 variants of uncertain significance (VUS) in the overall cohort (Tables 2, 3), which were assessed for quality by inspection of IGV read pile-ups or validated using Sanger sequencing (Supplementary Figure 1). Probands were deemed to be solved where a pathogenic or likely pathogenic variant was identified, while a subset of patients harbouring a VUS was deemed to be probably solved (*n* = 6, Figure 1). In these cases, there was insufficient pathogenic evidence for a confident pathogenic classification by ACMG criteria, no conflicting benign evidence (apart from computational predictors), and the proband's phenotype was consistent with the genetic findings. Notably, five of these six “probably solved” cases harboured a novel variant not previously implicated in disease and absent from 537 African-ancestry control genomes. There was no difference in the proportion of solved cases between the GN and HSP groups (44 vs. 48%, *p* = 0.7; Figure 1).

**Table 2:** Variants identified by whole exome and whole genome sequencing in probands with genetic neuropathies.

**Family ID**	**Sex**	**Anc**.	**AAO**	**Clinical features**	**Gene variant zygosity ClinVar accession(s)**	**Disease (MONDO ID) inheritance**	**ACMG classification with evidence codes**
**Solved cases**							
fam_006^a^	F	SAC	10	HMN plus, hyperreflexia	*MFN2* NP_055689.1:p.Thr206Ala het SCV003930375	CMT axonal type 2A2 (MONDO:0012231) AD	LP (PP3, PM1, PM2_supp, PM5)
fam_081^a^	M	SAC	24	HMN; Optic neuropathy	*MFN2* NP_055689.1:p.Arg259Cys het SCV003930377	CMT axonal type 2A2 (MONDO:0012231) AD	P (PP3_str, PM1, PM2_supp, PM5, PS4)
fam_083^a^	F	SAC	10	CMT2; pyramidal signs	*MFN2* NP_055689.1:p.Arg280His het SCV003930380	CMT axonal type 2A2 (MONDO:0012231) AD	P (PP1_str, PP3_mod, PM1, PM2_supp, PM5, PS3_supp, PS4)
ICGNMD_18^b^	M	SAC	14	CMT2	*MORC2* NP_001290185.1:p.Arg252Trp het SCV003930346	CMT axonal type 2Z (MONDO:0014736) AD	P (PP1_str, PP2, PP3, PM1, PM2_supp PS3_supp, PS4)
ICGNMD_16^b^	F	B	10	CMT2	*ATP1A1* NP_000692.2:p.Ile592Thr het SCV003852622	CMT axonal type 2DD (MONDO:0054833) AD	LP (PP1_supp, PP2, PP3_mod, PM1, PM2_supp, PS3_supp, PS4_supp)
ICGNMD_59^b^	F	W	11	CMT2 + median neuropathy	*GJB1* NP_000157.1:p.Arg22Ter het SCV003930347	CMT X-linked dominant 1 (MONDO:0010549) AD	P (PP1, PM1, PM2_supp, PS4, PVS1)
ICGNMD_17^b^	F	W	20	HMN plus	*ADPRS* NP_060295.1:p.Val335Gly hom SCV003930343	Neurodegeneration, childhood-onset, stress-induced, variable ataxia and seizures (MONDO:0100095) AR	LP (PP1_str, PM2_supp, PM3, PS3_supp)
ICGNMD_4^b^	F	SAC	3	Ataxic neuropathy plus	*ATM* NP_000042.3:p.Thr1743IIe hom SCV003930348	Ataxia telangiectasia (MONDO:0008840) AR	LP (PP3_mod, PM2_supp, PM3_str, PS3_supp)
**Probably solved cases**							
ICGNMD_15^b^	F	SAC	<10	CMT2	***GAN*** **NP_071324.1:p.Gln94Ter** and NP_071324.1:p.Pro315Leu c/het SCV003930344 SCV003930345	Giant axonal neuropathy (MONDO:0000128) AR	VUS (PM2_supp, PVS1) and VUS (PM2_supp, PS4_supp)
ICGNMD_6^b^	M	W	45	CMT mixed	***MPZ*** **NP_000521.2:p.Glu71Gly** het SCV003852624	CMT2I (MONDO:0011889) AD	VUS (PM1, PM2_supp, BP4)
**Unsolved cases with variants of uncertain significance**							
fam_007^a^ ICGNMD_7^a,b^ ICGNMD_9^a,b^	M F F	B B B	5 13 20	CMT2	*MPV17* NP_002428.1:p.Gln36Ter and NP_002428.1:p.Arg125Trp c/het SCV003930386 SCV003930388	CMT axonal type 2EE (MONDO:0032728) AR	VUS (PM2_supp, PVS1 not met^*^) and VUS (PM2_supp, PP3)

M, male; F, female; W, White or European-genetic ancestry; SAC, South African Coloured; B, Black African; I, Indian-ancestry; Anc, ancestry; AAO, age at onset (years).

a Refers to whole genome sequencing.

b Refers to whole exome sequencing.

Het, heterozygous; hom, homozygous; c/het, compound heterozygous, although such variants were not confirmed in trans; AD, autosomal dominant; AR, autosomal recessive; VUS, variant of uncertain significance; LP, likely pathogenic; P, pathogenic; sup, supporting; mod, moderate; str, strong.

Novel variants (not previously implicated in disease) are shown in bold. CMT, Charcot-Marie-Tooth. HMN plus refers to clinical sensory impairment but normal sensory nerve action potentials on electrophysiology. CMT2 refers to an axonal sensorimotor neuropathy (nerve conduction velocities (CVs) >45m/s) (20). CMT mixed refers to an overall axonal pattern, although some nerves showed CVs <38 m/s. The median neuropathy refers to delayed distal latency and a conduction block in the forearm (>20% drop between proximal and distal motor amplitudes).

* PVS1 not assigned as loss of gene function is not an established pathogenic mechanism for MPV17-related CMT2EE (the majority of MPV17 variants associated with CMT2EE are homozygous missense variants while the single reported homozygous non-frameshift deletion, p.Asp126_Tyr136del, removes <10% of the MPV17 protein).

**Table 3:** Variants identified by whole exome and whole genome sequencing in probands with hereditary spastic paraplegia and/or spastic ataxia.

**Family ID**	**Sex**	**Anc**.	**AAO**	**Clinical features**	**Gene variant zygosity ClinVar accession(s)**	**Disease (MONDO ID) inheritance**	**ACMG classification with evidence codes**
**Solved cases**							
fam_001^a^	F	SAC	6	cHSP	***KIF1A*** **NP_001230937.1:p.Ala255Asp** het SCV003930349	SPG30 (MONDO:0012476) AD	LP (PM2_supp, PP2, PP3_mod, PM1, PM5)
ICGNMD_62^b^	F	W	9	cHSP	***KIF1A*** **NP_001230937.1:p.Thr341Pro** het SCV003930336	SPG30 (MONDO:0012476) AD	LP (PM2_supp, PP2, PP3_strong, PM1)
fam_059^a^	F	B	10	HSP	***PCYT2*** **NP_002852.1:p.Gly228Arg** hom SCV004024560	SPG82 (MONDO:0032906) AR	LP (PP3_str, PM1, PM2_supp)
fam_111^a^	M	SAC	37	HSP	***PSEN1*** **NP_000012.1:p.Arg278Gly** het SCV003930350	Alzheimer's disease 3 (MONDO:0011913) AD	LP (PM2_supp, PM5, PP3_str)
GAJ1022^c^	F	W	25	HSP	*SPAST* NM_014946.4:c.1099-1G>A het SCV003930390	SPG4 (MONDO:0008438) AD	P (PP3, PM2_supp, PS4_supp, PVS1)
ICGNMD_29^b^	F	SAC	1	cHSP	*ATL1* NP_056999.2:p.Arg403Pro het SCV003930335	SPG3A (MONDO:0008437) AD	LP (PM2_supp, PS4_supp, PP3, PM1, PM6_supp)
ICGNMD_49^b^	F	W	10	cHSP	*SPG7* NP_003110.1:p.Ala510Val hom SCV003930338	SPG7 (MONDO:0011803) AR	P (PP3_mod, PS3_supp, PM3_very-str, PM2_supp)
ICGNMD_22^b^	M	W	10	cHSP	*SPG11* NP_079413.3:p.Leu1997MetfsTer60 and NP_079413.3:p.Glu1026ArgfsTer4 c/het SCV003930339 SCV003930340	SPG11 (MONDO:0011445) AR	P (PP1_mod, PM2_supp, PM3_str, PVS1) and P (PM2_supp, PM3_v_str, PP1_mod, PVS1)
ICGNMD_26^b^	M	W	5	cHSP	*SPG11* NP_079413.3:p.Leu1997MetfsTer60 hom SCV003930339	SPG11 (MONDO:0011445) AR	P (PP1_mod, PM2_supp, PM3_str, PVS1)
**Probably solved cases**							
fam_009^a^	F	SAC	20	cHSP	***SPG11*** **NP_079413.3:p.Leu438Pro** hom SCV003930361	SPG11 (MONDO:0011445) AR	VUS (PM2_supp, PP3)
ICGNMD_8^b^	M	I	5	spastic ataxia plus	***RFT1*** **NP_443091.1:p.Gly494Ser** hom SCV003930391	RFT1-congenital disorder of glycosylation (MONDO:0012783) AR	VUS (PP3, PM2_supp)
fam_195^a^	F	B	16	cHSP	*ALDH18A1* NP_002851.2:p.Val451Met hom SCV003930351	SPG9B (MONDO:0014702) AR	VUS (PM2_supp, PM1)
fam_122^a^	M	SAC	15	HSP	*CYP7B1* NP_004811.1:p.His285Leu and NP_004811.1:p.His401Arg c/het SCV003930362 SCV003930374	SPG5A (MONDO:0010047) AR	VUS (PM2_supp, PM3_supp) and VUS (PM2_supp, PM3_supp, PP3_mod)
**Unsolved cases with variants of uncertain significance**							
fam_142^b^	F	SAC	17	HSP	*ALDH18A1* NM_002860.4:c.809-1G>C het SCV003930389	SPG9B (MONDO:0011006) AR	VUS (PM2_supp, PVS1)

M, male; F, female; W, White or European-genetic ancestry; SAC, South African Coloured; B, Black African; I, Indian-ancestry; Anc, ancestry; AAO, age at onset (years).

a Refers to whole genome sequencing.

b Refers to whole exome sequencing.

c Refers to gene panel sequencing.

Het, heterozygous; hom, homozygous; c/het, compound heterozygous, although such variants were not confirmed in trans; AD, autosomal dominant; AR, autosomal recessive; VUS, variant of uncertain significance; LP, likely pathogenic; P, pathogenic; sup, supporting; mod, moderate; str, strong; v_str, very strong.

Novel variants (not previously implicated in disease) are shown in bold. HSP, hereditary spastic paraplegia; cHSP, complex HSP when additional neurological systems are also involved resulting in, for example, ataxia, neuropathy (by electrophysiology), amyotrophy legs, cognitive impairment, and neuropathy.

### 3.3. Genetic neuropathies

The GN group comprised 31 probands, including 21 with CMT2 (axonal), seven with HMN, and four probands clinically classified as “other.” The latter group consisted of an individual from an autosomal dominant (AD) inherited optic neuropathy family, a proband with AD inherited gait difficulty since infancy, flat feet, and a small fibre neuropathy, a case with a small fibre neuropathy, and a proband with childhood-onset ataxic neuropathy (without telangiectasia). The proportion of solved GN cases did not differ by ancestry (African 8/16, 50% vs. European 6/16, 38%, *p* = 0.8). Furthermore, the ages at onset between solved (median 13, IQR 10–21) and unsolved (median 15, IQR 9–20) cases were similar (*p* = 0.7). Of the 14 solved GN cases, 11 (79%) had heterozygous pathogenic variants (compatible with AD inheritance), and 3 (21%) had homozygous/compound heterozygous variants [compatible with autosomal recessive (AR) inheritance].

The genes harbouring pathogenic mutations differed by ancestry; as expected, *PMP22* duplications were a frequent cause of GN in the European-ancestry CMT cases (three of 11). These cases had typical CMT1 features, with symptoms of ankle instability as an adolescent or young adult, degrees of distal lower limb atrophy, and sensorimotor demyelinating neuropathy more prominent in the legs than arms. Three of the four probands with *PMP22* duplications had European ancestry, and one had African (identified as SAC) ancestry. However, *MFN2* mutations were the most common among those with African genetic ancestry (three of 10, Table 2).

#### 3.3.1. Solved cases by next-generation sequencing

##### 3.3.1.1. *MFN2* (autosomal dominant CMT2A2)

We identified two pathogenic missense *MFN2* variants (p.Arg280His, p.Arg259Cys) and a likely pathogenic *MFN2* p.Thr206Ala variant in three probands with African ancestry and CMT2 (axonal neuropathy; Table 2). The proband carrying the *MFN2* p.Arg280His variant presented at age 10 with CMT2 and spasticity in her legs. The *MFN2* p.Arg259Cys variant was found in a CMT2 proband who developed optic neuropathy in adulthood.

**Figure 2:**
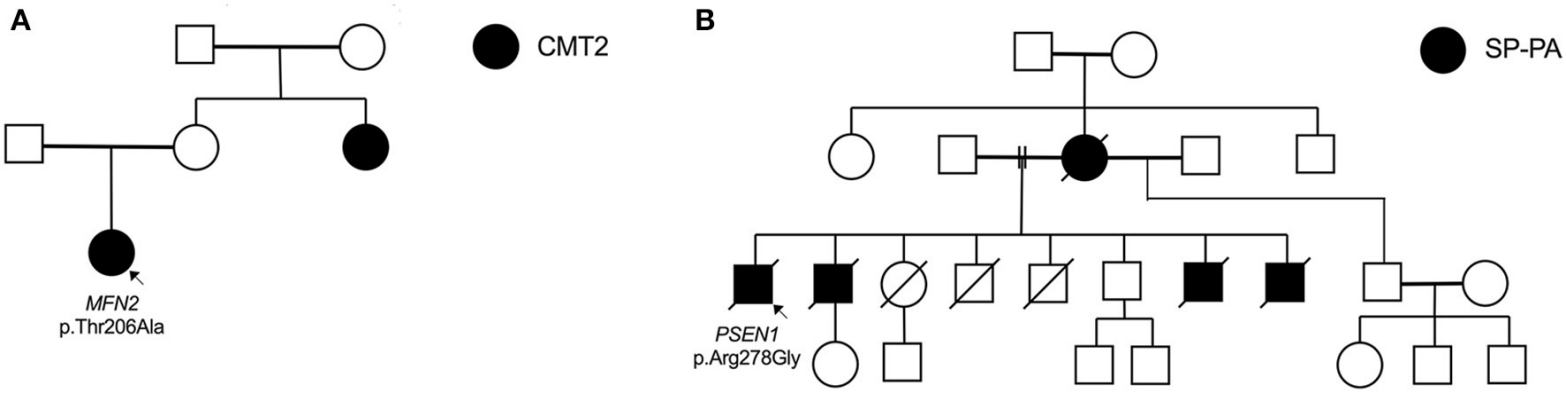
Selected pedigrees (genotyping only performed in probands). **(A)** Family of proband (fam_006) with heterozygous *MFN2* mutation. **(B)** Family of proband (fam_111) with novel *PSEN1* mutation presenting with spastic paraparesis (SP) followed by progressive aphasia (PA).

The proband carrying the *MFN2* p.Thr206Ala variant was diagnosed with childhood-onset CMT2 with prominent motor neuropathy and generalised hyperreflexia in the upper limbs and bulbar region. In her 4th decade, she required a wheelchair for mobility. At age 53, her mother was asymptomatic without clinical features, but her sister had moderately severe axonal sensorimotor neuropathy at age 31 (Figure 2A). Although rare cases of incomplete penetrance of *MFN2* pathogenic variants have been reported (21), at least three cases have been reported with symptom onset after the age of 53 years (22, 23) and with the variability of age at symptom onset within a family (24). The *MFN2* p.Thr206Ala variant occurs in the conserved dynamin-like GTPase domain together with other pathogenic variants, and the likely pathogenic *MFN2* p.Thr206Ile variant has been observed in multiple CMT2 probands (25).

##### 3.3.1.2. *MORC2* (autosomal dominant CMT2Z)

A pathogenic *MORC2* p.Arg252Trp variant was found in a proband without any family history (Table 2). During adolescence, the proband had trouble running, and the phenotype has progressed to resemble that of CMT2Z with sensory neuropathy, severe distal amyotrophy with distal greater than proximal weakness, truncal weakness, and type II respiratory failure requiring non-invasive nocturnal ventilation. Similar to our case, most CMT2Z patients are wheelchair-bound between their 20s and 40s. He displayed clinical “post-exertional” fasciculations in the legs and an axonal sensorimotor neuropathic process with active denervation.

##### 3.3.1.3. *ATP1A1* (autosomal dominant CMT2DD)

A likely pathogenic *ATP1A1* p.Ile592Thr variant was detected in an African-ancestry proband with an axonal length-dependent severe sensorimotor neuropathy with prominent peroneal muscular atrophy (foot drop) manifesting in adolescence with clumsy gait (Table 2). This variant was previously described in a three-generational American family (26).

##### 3.3.1.4. *GJB1* (x-linked dominant CMTX1)

A known pathogenic variant in *GJB1* p.Arg22Ter was identified in a woman with European ancestry who developed a clumsy gait at age 12 (Table 2). She was examined at 51 and again at 81. She has pes cavus, distal limb amyotrophy, Achilles' tendon contractures, moderately severe axonal length-dependent sensorimotor neuropathy (but median nerve conduction velocities were 37 m/s), and proximal hip girdle weakness. She has required foot orthoses and crutches for mobilisation since her seventh decade. The severity of her CMT is compatible with previously reported female carriers (27).

##### 3.3.1.5. *ADPRS* or *ADPRHL2* (autosomal recessive CONDSIAS presenting as hereditary motor neuropathy)

We identified a homozygous *ADPRS* p.Val335Gly variant in a European ancestry proband with severe distal HMN (Table 2). This rare variant was previously described in three unrelated probands (28, 29). This proband had severe axonal HMN with distal amyotrophy, proximal weakness, hyperreflexia (with extensor plantar responses at age 29), dysphagia, and increasing type II respiratory failure, from which she died in her fifth decade. Her brother manifested childhood-onset HMN and died in his third decade of complications secondary to a fall.

##### 3.3.1.6. *ATM* (autosomal recessive ataxia-telangiectasia)

This patient with early childhood-onset ataxic neuropathy (without telangiectasia) had a homozygous likely pathogenic *ATM* p.Thr1743Ile variant (Table 2). This variant was previously described in *trans* with other pathogenic *ATM* variants in a few European ataxia-telangiectasia probands (30, 31).

#### 3.3.2. Probably solved cases by next-generation sequencing

##### 3.3.2.1. *GAN* (autosomal recessive giant axonal neuropathy-1)

We detected biallelic *GAN* variants (novel p.Gln94Ter and known p.Pro315Leu) in an African-ancestry proband with CMT (Table 2). Our case has an affected sibling (not examined) but non-consanguineous parents. Apart from moderately severe sensorimotor axonal neuropathy and pes cavus, which started in childhood and progressed to severe proximal weakness, which required mobilising with a wheelchair in her fifth decade, she also has diaphragmatic weakness and autonomic nervous system involvement with hyperhidrosis and constipation. *GAN* encodes gigaxonin and missense variants, resulting in a phenotypic continuum ranging from severe (with central nervous system neurodegeneration) to a milder phenotype, such as our case (32).

##### 3.3.2.2. *MPZ* (autosomal dominant CMT2I)

Mutations in the *MPZ* gene result in autosomal dominant CMT, presenting as a phenotype spectrum from early onset severe CMT1 to late-onset CMT2. We identified a two-generation European ancestry family presenting with symptoms in middle age with the novel *MPZ* p.Glu71Gly variant (Table 2). Although this variant has not been reported, a pathogenic null variant (*MPZ* p.Glu71Ter) at the same amino acid location was described in a CMT family with incomplete penetrance (33). Furthermore, a VUS (because there was insufficient evidence to support its pathogenicity) at the same amino acid position (p.Glu71Lys) was detected in a CMT1 case (34).

#### 3.3.3. Unsolved cases

Eighteen GN cases (58%) remained without a genetic diagnosis (Figure 1), which included a screening of the *SPAST, SPG11, SPG7, MFN2, MPZ, KIF5A*, and *GJB1* genes for structural variants. Half of the unsolved cases were CMT (*n* = 10), of which nine were axonal neuropathies, and one had severe hypertrophic neuropathy. Five of the unsolved cases had HMN (ages at onset between 9 and 32 years and normal sensory nerve action potentials on electrophysiology together with evidence of motor axonopathy), and three had other neuropathies, including AD optic neuropathy, small fibre neuropathy, and AD infantile gait difficulty with pes planus and numbness (normal electrophysiology).

Of the nine unsolved probands with axonal neuropathies, five were Black African patients from the Western and Eastern Cape regions of South Africa who speak isiXhosa, with an average age of disease onset of 13 years (range 7–20). In all five of these CMT2 probands, we identified the heterozygous p.Gln36Ter *MPV17* variant (reported to be pathogenic for Navajo neurohepatopathy in homozygosity, ClinVar SCV000282035.1), and three of these cases also had an additional missense *MPV17* p.Arg125Trp variant (not confirmed in *trans*). Biallelic homozygous *MPV17* variants have been reported to result in juvenile-onset isolated peripheral sensorimotor neuropathy (CMT2EE) without hepatocerebral involvement, as occurs in autosomal recessive (AR) mitochondrial DNA depletion syndrome 6 (Navajo neurohepatopathy). The *MPV17* p.Gln36Ter variant has a minor allele frequency (MAF) >0.001 in two independent SAB control samples from Cape Town, South Africa: 11/1,500 alleles in newborns (MAF 0.007, one in 68 carrier frequency) (35) and 6/400 alleles in another study (MAF 0.015, one in 33 carrier frequency) (36) (personal communication L. Majara) and has not been found in Black individuals from other parts of South Africa (internal database). The p.Arg125Trp variant is reported in gnomAD v2.1.1 (MAF 0.0002) with the highest frequency in the African/African-American subpopulation, although our internal control database has an even higher frequency (MAF 0.005). In our internal control database, we have not identified the p.Gln36Ter and p.Arg125Trp variants in the same individual. However, only 58/537 (11%) control samples were ancestry-matched to the small geographical cluster of p.Gln36Ter carriers as determined by ancestry principal component analysis (data not shown). Further pathogenic evidence is required to assert that compound heterozygous *MPV17* p.Gln36Ter/p.Arg125Trp VUS variants explain a localised cluster of CMT2 in South Africa, particularly since a high regional prevalence of CMT2 has not been reported, the clinical phenotype among our three reported cases is dissimilar (Supplementary Table 3), there is a lack of ancestry-matched control data to determine the frequency of p.Gln36Ter/p.Arg125Trp co-occurrence in controls and the strength of the evidence supporting the *MPV17*-CMT2EE gene-disease dyad have not yet been evaluated by a ClinGen gene curation expert panel.

### 3.4. Hereditary spastic paraplegia

Of the 29 cases with HSP or spastic ataxias, 48% were solved. The median age of onset was 15 years (IQR 9–24) for HSP and 15 years (IQR 6–25) for spastic ataxias (Table 1). The proportion of solved HSP cases did not differ by ancestry (African 9/20, 45% vs. European 5/9, 56%, *p* = 0.5). Furthermore, the ages at onset between resolved (median 13, IQR 8–24) and unresolved (median 16, IQR 9–28) cases were similar (*p* = 0.3). In an African ancestry case with spastic ataxia and without a family history, a heterozygous *ATXN1* trinucleotide expansion was detected to designate SCA1. Of the 14 solved HSP cases, six (43%) had heterozygous pathogenic variants (compatible with AD inheritance), and eight (57%) had homozygous/compound heterozygous variants (compatible with AR inheritance).

Among the African-ancestry probands, variants in rare HSP genes such as *PCYT2* and *PSEN1* were identified, whereas the European-ancestry probands harboured variants in common HSP genes: *SPG11* (*n* = 2/5), *SPAST* (*n* = 1/5), *SPG7* (*n* = 1/5) and a novel variant in *KIF1A* (*n* = 1/5; Table 3).

#### 3.4.1. Solved cases by next-generation sequencing

##### 3.4.1.1. *KIF1A* (autosomal dominant SPG30)

We detected two AD-HSP30 probands in our cohort harbouring novel variants in the *KIF1A* mutational hotspot region in the motor domain (amino acids 1–361) (37) (Table 3). A likely pathogenic *KIF1A* p.Ala255Asp variant was identified in a mother and daughter with a complicated HSP phenotype with symptom onset in childhood and additional cerebellar dysfunction (gait ataxia, horizontal and downbeat nystagmus), mild intellectual impairment, optic neuropathy, and a sensorimotor axonal neuropathy with acral leg ulceration. These are common features in HSP30 (37, 38). A second likely pathogenic *KIF1A* p.Thr341Pro variant was detected in a proband whose clinical features fit those of HSP30.

##### 3.4.1.2. *PCYT2* (autosomal recessive SPG82)

We detected a novel homozygous *PCYT2* p.Gly246Arg variant in a Black individual with complex HSP, optic neuropathy, and length-dependent sensorimotor neuropathy (Table 3). She manifested gait unsteadiness in childhood, progressive optic neuropathy since adolescence and spastic dysarthria and seizures in adulthood. She was completely blind in middle age, which underscores the prominent role of PCYT2 in ocular homeostasis via the CDP-ethanolamine pathway (39).

##### 3.4.1.3. *PSEN1* (autosomal dominant Alzheimer's disease 3)

A novel *PSEN1* p.Arg278Gly pathogenic variant was found in an AD African ancestry family (mother and four sons) presenting with progressive spastic paraparesis starting in their fourth decade and followed within months by progressive non-fluent aphasia (and later frontal dysexecutive dysfunction; Table 3, Figure 2B). The condition progressed to akinetic mutism and death over 8 to 10 years from symptom onset.

##### 3.4.1.4. *SPAST* (autosomal dominant SPG4)

Although a common *SPAST* c.1099-1G>A variant was found in a European-ancestry proband (Table 3), which accounts for 60%−80% of AD-HSP cases in European and Asian cohorts (40, 41), we did not find *SPAST* variants (including structural variants) in our African-ancestry patients (*n* = 8 with HSP) even after visual inspection of WGS data on IGV and by using ClinSV.

##### 3.4.1.5. *ATL1* (autosomal dominant SPG3A)

We detected a likely pathogenic *ATL1* p.Arg403Pro variant in the exon 12 mutational hotspot (42) in an individual with early childhood-onset HSP (Table 3). This is the second most common AD-HSP in the global north/European cohorts (43).

##### 3.4.1.6. *SPG7* (autosomal recessive SPG7)

The common homozygous *SPG7* p.Ala510Val pathogenic variant was detected in a European-ancestry individual (Table 3). Although this variant has a high population frequency on gnomAD (gnomAD v2 exomes 0.48%/gnomAD v3 genomes 0.61% in the European non-Finnish population), it is found to segregate with disease in multiple families and is described as a common founder mutation in *SPG7* cases of British ancestry (44).

##### 3.4.1.7. *SPG11* (autosomal recessive SPG11)

SPG11 is the most common subtype of autosomal recessive HSP with cognitive impairment (45). Two pathogenic *SPG11* mutations, homozygous *SPG11* p.Leu1997MetfsTer60, and compound heterozygous variants, p.Leu1997MetfsTer60 and p.Glu1026ArgfsTer4, were detected in two probands (46, 47) (Table 3).

#### 3.4.2. Probably solved cases by next-generation sequencing

##### 3.4.2.1. *SPG11* (autosomal recessive SPG11)

A novel homozygous missense *SPG11* p.Leu438Pro variant was curated as a good disease candidate in an African ancestry (SAC) proband (Table 3). Although loss of function variants have been implicated in SPG11 disease, there are reports in the literature of SPG11 probands with homozygous missense *SPG11* variants (48, 49).

##### 3.4.2.2. *RFT1* (autosomal recessive congenital disorder of glycosylation)

**Figure 3:**
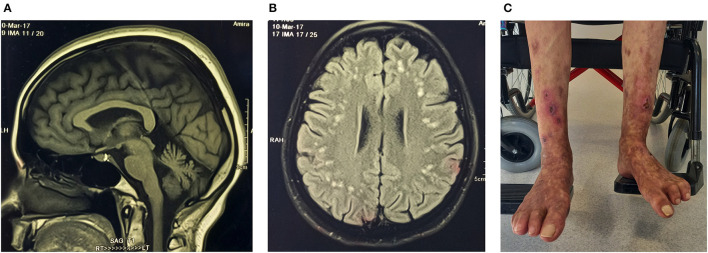
Proband (ICGNMD_8) with a milder phenotype of RFT1-congenital disorder of glycosylation. Brain imaging at age 35 years. **(A)** Sagittal T1-weighted MRI-brain scan showing cerebellar atrophy. **(B)** An axial brain FLAIR MRI image showing widespread discrete high signal intensity lesions in the subcortical white matter. Apart from the RFT1-disorders known to have coagulation factor abnormalities, the patient had no other cardiovascular risk factors to account for these white matter abnormalities of presumed vascular origin. **(C)** Spontaneous painful lesions with subsequent ulceration, which occurred in the distal extremities (before anticoagulation). Since oral anticoagulants were started for possible clotting abnormalities as a cause, the lesions have disappeared in the forearms and are substantially less in the legs (age 49).

A homozygous missense variant p.Gly494Ser in exon 13 of the *RFT1* gene was classified as a VUS for RFT1-congenital disorder of glycosylation (CDG) with autosomal recessive inheritance (Table 3). The proband is of Indian ancestry, and her parents were consanguineous. RFT1-CDG was first recognised as a severe congenital disorder of N-linked glycosylation characterised by developmental delay, failure to thrive, myoclonic encephalopathy, seizures, and sensorineural hearing loss. However, a milder variant CDG phenotype was reported, resembling our patient's phenotype (50, 51). Our patient had a childhood-onset intellectual disability, adult-onset cerebellar ataxia, spasticity, mild sensorineural hearing impairment, inverted nipples, motor > sensory length-dependent axonal polyneuropathy, and spontaneous painful ulcers developing in the distal leg > forearm (Figure 3). Her brain MR imaging at age 35 showed periventricular white matter signal hyperintensities on FLAIR imaging and cerebellar atrophy. RFT1-CDG probands are known to suffer from clotting abnormalities. Interestingly, despite a negative clotting dysfunction screen, this proband's spontaneous, painful, and ulcerating skin lesions in her extremities improved substantially on oral anticoagulant therapy.

##### 3.4.2.3. *ALDH18A1* (autosomal recessive SPG9B)

*ALDH18A1* is known to result in SPG9A (autosomal dominant) and SPG9B (autosomal recessive) HSP (52). A homozygous missense *ALDH18A1* p.Val451Met variant was found in an African-ancestry proband presenting with gait difficulties in adolescence (Table 3). At age 36, the patient manifested, in addition to severe spasticity of the legs, distal amyotrophy, and pes cavus, sensory neuropathy, spasticity of the bulbar region, as well as mild cerebellar signs with nystagmus and past-pointing in the arms. This is the first report of SPG9B in sub-Saharan Africa, with previous reports in European and Japanese populations (53, 54). The majority of these missense variants occur in the L-glutamyl-5-phosphate reductase (G5PR) domain, where the p.Val451Met variant is also located.

##### 3.4.2.4. *CYP7B1* (autosomal recessive SPG5A)

Biallelic *CYP7B1* p.His401Arg and p.His285Leu variants were detected in a proband with HSP symptom onset in adolescence and accompanied by a substantial proprioceptive loss in his legs (Table 3). The p.His401Arg variant was previously described in a sporadic Taiwanese SPG5A case with another pathogenic *CYP7B1* variant (p.Arg112Ter) (55). The second *CYP7B1* p.His285Leu variant was previously detected in a homozygous state in an Italian SPG5A proband (56). Biallelic variants in the *CYP7B1* gene account for <10% of all AR HSP in European and Asian populations (57).

#### 3.4.3. Unsolved cases

Among the HSP cases, 52% remained without a genetic diagnosis, irrespective of genetic ancestry, including 11 with HSP and four with spastic ataxia (Figure 1). Searching for structural variants in the WES/WGS data of unsolved cases did not identify any additional disease candidates in the HSP and spastic ataxia genes.

We curated an *ALDH18A1* c.809-1G>C splice variant in a proband with gait difficulties since adolescence as a variant of uncertain significance for SPG9B (Table 3) (52). Although this variant is rare in gnomAD, it has a MAF of 0.032% (32/100,000 alleles) in an internal database from the Netherlands (personal communication, E.-J. Kamsteeg). It has been identified in at least three probands by clinical testing (ClinVar SCV001929264.1, SCV001952732.1, and SCV001970262.1) and is classified as likely pathogenic for AR HSP (in each proband, the variant was confirmed in trans with another *ALDH18A1* VUS and the parents were unaffected heterozygous carriers). In our proband's WGS data, we have not identified a second candidate *ALDH18A1* single nucleotide variant or structural variant consistent with this variant's reported role in SPG9B with autosomal recessive inheritance.

## 4. Discussion

This report describes the mutational profile of inherited neuropathies, spastic paraplegias, and spastic ataxias in a small but first cohort from South Africa comprising 61 probands. Among the European-ancestry cases, despite the small sample size, the profile was similar to that expected for European probands for both the genetic neuropathies and the HSP cases. In our cohort, among the GN group, the notable difference was the lack of CMT1A-*PMP22* cases among Black South Africans, whereas this gene accounts for 37% of European cohorts (58). In the HSP group, the difference was the lack of *SPG4, SPG7*, and *SPG11* variants in African-ancestry probands, representing the common AD and AR genotypes in Europeans and Asians (59, 60). The prevalence of HSP genetic subtypes varies by geography, reflecting isolation effects (increased risk of AD-HSP in Norwegian and Sardinian populations attributed to founder effects) (40) and consanguinity (increased risk of AR-HSP in North Africa and Western Asia) (2, 40), neither of which are characteristic of African ancestry subpopulations in South Africa. While referral bias is unlikely to explain this different mutational profile, these findings require validation in a larger sample.

In this cohort, the solved rate among the genetic neuropathies (44%) and the HSP and spastic ataxia group (48%) was similar. The proportion of HSP/spastic ataxia probands achieving a genetic diagnosis was similar to a larger European HSP cohort (49%) (61). However, in this study, the solved rate of GN probands was lower than a larger European GN cohort (~60%), likely due to fewer CMT1 cases, which comprised 55% of that cohort (58).

While the study's aims did not include investigating novel disease genes, 8/28 (29%) resolved probands harbouring a novel disease-causing variant in a known disease gene. This highlights the importance of studying GN and HSP cohorts of diverse and underrepresented ancestries and sharing their pathogenic variants with the wider scientific community via databases such as ClinVar to enhance genetic diagnosis of these conditions worldwide.

The use of population descriptors in this study has only been employed insofar as to guide the analysis of the genomic data (62). African genomes are highly diverse, and African-ancestry individuals from South Africa are not represented in the gnomAD database, the cornerstone of population frequency information when curating variants for rare diseases. Necessarily, our study leveraged allele frequency information from an internal sub-Saharan African ancestry control dataset (*n* = 1,074 alleles) to guide the curation of candidate variants absent from gnomAD. While this is a substantial continental sample of non-gnomAD ancestry representation, the high levels of diversity and population substructure in Africa will likely require even greater numbers of ancestry-matched controls by country subregion to derive accurate allele frequency information for African subpopulations. This is illustrated by the high regional frequency (MAF > 1%) of the *MPV17* p.Gln36Ter variant in South Africa, despite its absence from gnomAD. As genomic research for rare diseases expands in Africa, acknowledging the ongoing importance of case-control study designs and encouraging concerted efforts to share aggregate African frequency data (63) are necessary to avoid misclassification of variant pathogenicity (64). Indeed, the value of studying African genomes to drive the equitable benefit of genomic medicine for rare diseases will not be realised unless African genomic data is shared with the scientific community. This will inform current efforts to evaluate disease-gene validity and define pathogenic disease variants, ultimately guiding gene-based therapy development and clinical trial eligibility.

In this report, we provided confirmatory evidence of phenotype expansion for three disease entities: autosomal recessive CONDSIAS manifesting as hereditary motor neuropathy with hyperreflexia due to a homozygous *ADPRS* variant; autosomal recessive giant axonal neuropathy presenting as a non-classical early-onset motor and sensory neuropathy due to biallelic *GAN* variants; and autosomal recessive congenital disorder of glycosylation due to a homozygous *RFT1* variant presenting without seizures. These cases were solved through close collaboration between the referring clinician and genomic analysts and highlighted the limitations of using human phenotype ontology terms to direct clinical interpretation of genomic data for genetic diagnosis. Indeed, even beyond phenotype expansion within specific disease entities, as demonstrated by the aforementioned examples, it is increasingly recognised that genetic neuropathies and hereditary spastic paraplegias (grouped separately for this report) may represent a disease spectrum due to their overlapping clinical and genetic features. Therefore, a broad approach to the genetic investigation of these disorders is warranted, while prompt sharing of variant interpretations from Africa will ensure that the ClinGen gene curation expert panel's efforts to assess the evidence of gene-disease relationships for this group of disorders consider global genetic diversity. A case in point is our observation of compound heterozygous *MPV17* variants in SA Black probands with CMT2. These case reports, which have been deposited in ClinVar, will now be considered when the genetic evidence for *MPV17* variants and CMT2EE is objectively reviewed.

While genetic testing to diagnose NMDs is now the standard of care in developed countries to achieve a definitive molecular diagnosis, the South African probands described in this report were able to access exome and genome sequencing only through their participation in research studies. Although the sequencing data was generated largely by international consortia outside of South Africa, it is noteworthy that the raw data for this report were analysed and interpreted locally as a critical exercise in capacity building, which is essential for the future implementation of local NGS-based genetic testing strategies.

The proportion of our cohort that remains without a genetic diagnosis is similar to other population cohorts, suggesting a common biological, genetic mechanism underlying this missing heritability, such as noncoding genetic variants, complex structural changes, or epigenetic changes (61). Such classes of genetic variation are not adequately examined by short-read approaches, and long-read whole genome sequencing is expected to drive disease gene discovery for these disorders in the future.

## 5. Conclusion

This first cohort analysis from a South African neurology clinic using whole exome and whole genome sequencing data suggests that the mutational profile of both genetic neuropathies and hereditary spastic paraplegias (and spastic ataxias) is different in subjects with African ancestry compared to those with European ancestry. This highlights the importance of including genetically diverse cohorts in research programs with the ultimate goal of therapy development.

## Data availability statement

The datasets presented in this study have been deposited in the ClinVar repository with the following accession numbers: SCV003930375, SCV003930377, SCV003930380, SCV003930346, SCV003852622, SCV003930347, SCV003930343, SCV003930348, SCV003930344, SCV003930345, SCV003852624, SCV003930386, SCV003930388, SCV003930349, SCV003930336, SCV003930350, SCV003930390, SCV003930335, SCV003930338, SCV003930339, SCV003930340, SCV003930361, SCV003930391, SCV003930351, SCV003930362, SCV003930374, SCV003930389, and SCV004024560.

## Ethics statement

The studies involving humans were approved by University of Cape Town Human Research Ethics Committee. The studies were conducted in accordance with the local legislation and institutional requirements. Written informed consent for participation in this study was provided by the participants' legal guardians/next of kin. Written informed consent was obtained from the individual(s), and minor(s)' legal guardian/next of kin, for the publication of any potentially identifiable images or data included in this article.

## Author contributions

AM analysed the NGS data and wrote the first draft of the manuscript. LW, NF, and KN provided organisational support. JV and GW provided bioinformatics support. CR and MR assisted with data interpretation for ICGNMD cases. MB leads CReATe and assists with data interpretation for CReATe cases. JH, ES, SR, and JW assisted with patient recruitment and clinical phenotyping. JW and JH are ICGNMD PIs and recruited cases from South Africa. MH leads the ICGNMD. MN and JH contributed to the conception, design, and supervision of the study (genomics and clinical phenotyping, respectively). All authors contributed to the manuscript's revision, read, and approved the submitted version.

## References

[1] Nel M Mahungu AC Monnakgotla N Botha GR Mulder NJ Wu G. Revealing the mutational spectrum in Southern Africans with amyotrophic lateral sclerosis. Neurol Genet. (2022) 8:e654. 10.1212/NXG.0000000000000654 35047667 PMC8756565

[2] Mahungu AC Monnakgotla N Nel M Heckmann JM. A review of the genetic spectrum of hereditary spastic paraplegias, inherited neuropathies and spinal muscular atrophies in Africans. Orphanet J Rare Dis. (2022) 17:1–12. 10.1186/s13023-022-02280-2 35331287 PMC8944057

[3] Rossor AM Evans MRB Reilly MM. A practical approach to the genetic neuropathies. Pract Neurol. (2015) 15:187–98. 10.1136/practneurol-2015-001095 25898997

[4] Sengupta D Choudhury A Fortes-Lima C Aron S Whitelaw G Bostoen K. Genetic substructure and complex demographic history of South African Bantu speakers. Nat Commun. (2021) 12:2080. 10.1038/s41467-021-22207-y 33828095 PMC8027885

[5] Choudhury A Aron S Botigué LR Sengupta D Botha G Bensellak T. High-depth African genomes inform human migration and health. Nature. (2020) 586:741–8. 10.1038/s41586-020-2859-7 33116287 PMC7759466

[6] Mallick S Li H Lipson M Mathieson I Gymrek M Racimo F. The Simons Genome Diversity Project: 300 genomes from 142 diverse populations. Nature. (2016) 538:201–6. 10.1038/nature18964 27654912 PMC5161557

[7] Choudhury A Ramsay M Hazelhurst S Aron S Bardien S Botha G. Whole-genome sequencing for an enhanced understanding of genetic variation among South Africans. Nat Commun. (2017) 8:1–12. 10.1038/s41467-017-00663-9 29233967 PMC5727231

[8] Nel M Mulder N Europa TA Heckmann JM. Using whole genome sequencing in an African subphenotype of myasthenia gravis to generate a pathogenetic hypothesis. Front Genet. (2019) 10:136. 10.3389/fgene.2019.00136 30881381 PMC6406016

[9] Nel M Agenbag GM Henning F Cross HM Esterhuizen A Heckmann JM. *C9orf72* repeat expansions in South Africans with amyotrophic lateral sclerosis. J Neurol Sci. (2019) 401:51–4. 10.1016/j.jns.2019.04.026 31009932 PMC6556408

[10] Schatz MC Philippakis AA Afgan E Banks E Carey VJ Carroll RJ. Inverting the model of genomics data sharing with the NHGRI genomic data science analysis, visualization, and informatics lab-space. Cell Genom. (2022) 2:100085. 10.1016/j.xgen.2021.100085 35199087 PMC8863334

[11] Pais LS Snow H Weisburd B Zhang S Baxter SM DiTroia S. seqr: a web-based analysis and collaboration tool for rare disease genomics. Hum Mutat. (2022) 43:698–707. 10.1002/humu.24366 35266241 PMC9903206

[12] Martin AR Williams E Foulger RE Leigh S Daugherty LC Niblock O. PanelApp crowdsources expert knowledge to establish consensus diagnostic gene panels. Nat Genet. (2019) 51:1560–5. 10.1038/s41588-019-0528-2 31676867

[13] Preston CG Wright MW Madhavrao R Harrison SM Goldstein JL Luo X. ClinGen variant curation interface: a variant classification platform for the application of evidence criteria from ACMG/AMP guidelines. Genome Med. (2022) 14:1–12. 10.1186/s13073-021-01004-8 35039090 PMC8764818

[14] Richards S Aziz N Bale S Bick D Das S Gastier-Foster J. Standards and guidelines for the interpretation of sequence variants: a joint consensus recommendation of the American College of Medical Genetics and Genomics and the Association for Molecular Pathology. Genet Med. (2015) 17:405–24. 10.1038/gim.2015.30 25741868 PMC4544753

[15] Harrison SM Biesecker LG Rehm HL. Overview of specifications to the ACMG/AMP variant interpretation guidelines. Curr Protoc Hum Genet. (2019) 103:e93. 10.1002/cphg.93 31479589 PMC6885382

[16] Chunn LM Nefcy DC Scouten RW Tarpey RP Chauhan G Lim MS. Mastermind: a comprehensive genomic association search engine for empirical evidence curation and genetic variant interpretation. Front Genet. (2020) 11:577152. 10.3389/fgene.2020.577152 33281875 PMC7691534

[17] Minoche AE Lundie B Peters GB Ohnesorg T Pinese M Thomas DM. ClinSV: clinical grade structural and copy number variant detection from whole genome sequencing data. Genome Med. (2021) 13:1–19. 10.1186/s13073-021-00841-x 33632298 PMC7908648

[18] Robinson JT Thorvaldsdóttir H Winckler W Guttman M Lander ES Getz G. Integrative genomics viewer. Nat Biotechnol. (2011) 29:24–6. 10.1038/nbt.1754 21221095 PMC3346182

[19] Belyeu JR Chowdhury M Brown J Pedersen BS Cormier MJ Quinlan AR. Samplot: a platform for structural variant visual validation and automated filtering. Genome Biol. (2021) 22:161. 10.1186/s13059-021-02380-5 34034781 PMC8145817

[20] Bird TD. Charcot-marie-tooth hereditary neuropathy overview. In: Adam MP Mirzaa GM Pagon RA Wallace SE Bean LJH Gripp KW Amemiya A, editors. GeneReviews®. Seattle, WA: University of Washington (1993–2023). 20301532

[21] Lawson VH Graham BV Flanigan KM. Clinical and electrophysiologic features of CMT2A with mutations in the mitofusin 2 gene. Neurology. (2005) 65:197–204. 10.1212/01.wnl.0000168898.76071.70 16043786

[22] Ma Y Sun A Zhang Y Fan D Liu X. The genotype and phenotype features in a large Chinese *MFN2* mutation cohort. Front Neurol. (2021) 12:757518. 10.3389/fneur.2021.757518 34721278 PMC8548668

[23] Abati E Manini A Velardo D Del Bo R Napoli L Rizzo F. Clinical and genetic features of a cohort of patients with *MFN2*-related neuropathy. Sci Rep. (2022) 12:6181. 10.1038/s41598-022-10220-0 35418194 PMC9008012

[24] Del Bo R Moggio M Rango M Bonato S D'Angelo MG Ghezzi S. Mutated mitofusin 2 presents with intrafamilial variability and brain mitochondrial dysfunction. Neurology. (2008) 71:1959–66. 10.1212/01.wnl.0000327095.32005.a4 18946002

[25] Sitarz KS Yu-Wai-Man P Pyle A Stewart JD Rautenstrauss B Seeman P. *MFN2* mutations cause compensatory mitochondrial DNA proliferation. Brain. (2012) 135:e219–e219. 10.1093/brain/aws049 22492563 PMC3407419

[26] Lassuthova P Rebelo AP Ravenscroft G Lamont PJ Davis MR Manganelli F. Mutations in *ATP1A1* cause dominant Charcot-Marie-Tooth type 2. Am J Hum Genet. (2018) 102:505–14. 10.1016/j.ajhg.2018.01.023 29499166 PMC5985288

[27] Mandich P Grandis M Geroldi A Acquaviva M Varese A Gulli R. Gap junction beta 1 (*GJB1*) gene mutations in Italian patients with X-linked Charcot-Marie-Tooth disease. J Hum Genet. (2008) 53:529–33. 10.1007/s10038-008-0280-4 18379723

[28] Beijer D Agnew T Rack JGM Prokhorova E Deconinck T Ceulemans B. Biallelic *ADPRHL2* mutations in complex neuropathy affect ADP ribosylation and DNA damage response. Life Sci Alliance. (2021) 4:e202101057. 10.26508/lsa.202101057 34479984 PMC8424258

[29] Danhauser K Alhaddad B Makowski C Piekutowska-Abramczuk D Syrbe S Gomez-Ospina N. Bi-allelic *ADPRHL2* mutations cause neurodegeneration with developmental delay, ataxia, and axonal neuropathy. Am J Hum Genet. (2018) 103:817–25. 10.1016/j.ajhg.2018.10.005 30401461 PMC6218634

[30] Mandola AB Reid B Sirror R Brager R Dent P Chakroborty P. Ataxia telangiectasia diagnosed on newborn screening–case cohort of 5 years' experience. Front Immunol. (2019) 10:2940. 10.3389/fimmu.2019.02940 31921190 PMC6932992

[31] Stankovic T Kidd AMJ Sutcliffe A McGuire GM Robinson P Weber P. *ATM* mutations and phenotypes in ataxia-telangiectasia families in the british isles: expression of mutant ATM and the risk of leukemia, lymphoma, and breast cancer. Am J Hum Genet. (1998) 62:334–45. 10.1086/301706 9463314 PMC1376883

[32] Aharoni S Barwick KES Straussberg R Harlalka GV Nevo Y Chioza BA. Novel homozygous missense mutation in *GAN* associated with Charcot-Marie-Tooth disease type 2 in a large consanguineous family from Israel. BMC Med Genet. (2016) 17:82. 10.1186/s12881-016-0343-x 27852232 PMC5112725

[33] Lagueny A Latour P Vital A Le Masson G Rouanet M Ferrer X. Mild recurrent neuropathy in CMT1B with a novel nonsense mutation in the extracellular domain of the *MPZ* gene. J Neurol Neurosurg Psychiatry. (2001) 70:232–5. 10.1136/jnnp.70.2.232 11160475 PMC1737217

[34] Subréville M Bonello-Palot N Yahiaoui D Beloribi-Djefaflia S Fernandes S Stojkovic T. Genotype–phenotype correlation in French patients with myelin protein zero gene-related inherited neuropathy. Eur J Neurol. (2021) 28:2913–21. 10.1111/ene.14948 34060176

[35] Meldau S De Lacy RJ Riordan GTM Goddard EA Pillay K Fieggen KJ. Identification of a single *MPV17* nonsense-associated altered splice variant in 24 South African infants with mitochondrial neurohepatopathy. Clin Genet. (2018) 93:1093–6. 10.1111/cge.13208 29318572

[36] Stevenson A Akena D Stroud RE Atwoli L Campbell MM Chibnik LB. Neuropsychiatric genetics of african populations-psychosis (NeuroGAP-psychosis): a case-control study protocol and GWAS in Ethiopia, Kenya, South Africa and Uganda. BMJ Open. (2019) 9:e025469. 10.1136/bmjopen-2018-025469 30782936 PMC6377543

[37] Pennings M Schouten MI van Gaalen J Meijer RPP de Bot ST Kriek M. *KIF1A* variants are a frequent cause of autosomal dominant hereditary spastic paraplegia. Eur J Hum Genet. (2020) 28:40–9. 10.1038/s41431-019-0497-z 31488895 PMC6906463

[38] Klebe S Lossos A Azzedine H Mundwiller E Sheffer R Gaussen M. *KIF1A* missense mutations in SPG30, an autosomal recessive spastic paraplegia: distinct phenotypes according to the nature of the mutations. Eur J Hum Genet. (2012) 20:645–9. 10.1038/ejhg.2011.261 22258533 PMC3355258

[39] Vaz FM McDermott JH Alders M Wortmann SB Kölker S Pras-Raves ML. Mutations in *PCYT2* disrupt etherlipid biosynthesis and cause a complex hereditary spastic paraplegia. Brain. (2019) 142:3382–97. 10.1093/brain/awz291 31637422 PMC6821184

[40] Vander Stichele G Durr A Yoon G Schüle R Blackstone C Esposito G. An integrated modelling methodology for estimating global incidence and prevalence of hereditary spastic paraplegia subtypes SPG4, SPG7, SPG11, and SPG15. BMC Neurol. (2022) 22:115. 10.1186/s12883-022-02595-4 35331153 PMC8944001

[41] Tesson C Koht J Stevanin G. Delving into the complexity of hereditary spastic paraplegias: how unexpected phenotypes and inheritance modes are revolutionizing their nosology. Hum Genet. (2015) 134:511–38. 10.1007/s00439-015-1536-7 25758904 PMC4424374

[42] Namekawa M Muriel M-P Janer A Latouche M Dauphin A Debeir T. Mutations in the *SPG3A* gene encoding the GTPase atlastin interfere with vesicle trafficking in the ER/Golgi interface and Golgi morphogenesis. Mol Cell Neurosci. (2007) 35:1–13. 10.1016/j.mcn.2007.01.012 17321752

[43] Zhao G Liu X. Clinical features and genotype-phenotype correlation analysis in patients with *ATL1* mutations: a literature reanalysis. Transl Neurodegener. (2017) 6:9. 10.1186/s40035-017-0079-3 28396731 PMC5379717

[44] Roxburgh RH Marquis-Nicholson R Ashton F George AM Lea RA Eccles D. The pAla510Val mutation in the SPG7 (paraplegin) gene is the most common mutation causing adult onset neurogenetic disease in patients of British ancestry. J Neurol. (2013) 260:1286–94. 10.1007/s00415-012-6792-z 23269439

[45] Stevanin G Santorelli FM Azzedine H Coutinho P Chomilier J Denora PS. Mutations in *SPG11*, encoding spatacsin, are a major cause of spastic paraplegia with thin corpus callosum. Nat Genet. (2007) 39:366–72. 10.1038/ng1980 17322883

[46] Pereira MC Loureiro JL Pinto-Basto J Brandão E Lopes AM Neves G. Alu elements mediate large *SPG11* gene rearrangements: further spatacsin mutations. Genet Med. (2012) 14:143–51. 10.1038/gim.2011.7 22237444

[47] Paisan-Ruiz C Dogu O Yilmaz A Houlden H Singleton A. *SPG11* mutations are common in familial cases of complicated hereditary spastic paraplegia. Neurology. (2008) 70(Issue 16, Part 2):1384–9. 10.1212/01.wnl.0000294327.66106.3d 18337587 PMC2730021

[48] Lynch DS Koutsis G Tucci A Panas M Baklou M Breza M. Hereditary spastic paraplegia in Greece: characterisation of a previously unexplored population using next-generation sequencing. Eur J Hum Genet. (2016) 24:857–63. 10.1038/ejhg.2015.200 26374131 PMC4688955

[49] Li C Yan Q Duan F Zhao C Zhang Z Du Y. Mild cognitive impairment in novel *SPG11* mutation-related sporadic hereditary spastic paraplegia with thin corpus callosum: case series. BMC Neurol. (2021) 21:12. 10.1186/s12883-020-02040-4 33430805 PMC7798194

[50] Ondruskova N Vesela K Hansikova H Magner M Zeman J Honzik T. RFT1-CDG in adult siblings with novel mutations. Mol Genet Metab. (2012) 107:760–2. 10.1016/j.ymgme.2012.10.002 23111317

[51] Quelhas D Jaeken J Fortuna A Azevedo L Bandeira A Matthijs G. RFT1-CDG: absence of epilepsy and deafness in two patients with novel pathogenic variants. JIMD Rep. (2019) 43:111–6. 10.1007/8904_2018_112 29923091 PMC6323008

[52] Coutelier M Goizet C Durr A Habarou F Morais S Dionne-Laporte A. Alteration of ornithine metabolism leads to dominant and recessive hereditary spastic paraplegia. Brain. (2015) 138:2191–205. 10.1093/brain/awv143 26026163 PMC4553756

[53] Kalmár T Maróti Z Zimmermann A Sztriha L. Tremor as an early sign of hereditary spastic paraplegia due to mutations in *ALDH18A1*. Brain Dev. (2021) 43:144–51. 10.1016/j.braindev.2020.07.015 32798076

[54] Wei Q Dong H-L Pan L-Y Chen C-X Yan Y-T Wang R-M. Clinical features and genetic spectrum in Chinese patients with recessive hereditary spastic paraplegia. Transl Neurodegener. (2019) 8:19. 10.1186/s40035-019-0157-9 31289639 PMC6593507

[55] Lan M-Y Yeh T-H Chang Y-Y Kuo H-C Sun HS Lai S-C. Clinical and genetic analysis of Taiwanese patients with hereditary spastic paraplegia type 5. Eur J Neurol. (2015) 22:211–4. 10.1111/ene.12407 24641183

[56] Arnoldi A Crimella C Tenderini E Martinuzzi A D'Angelo M Musumeci O. Clinical phenotype variability in patients with hereditary spastic paraplegia type 5 associated with *CYP7B1* mutations. Clin Genet. (2012) 81:150–7. 10.1111/j.1399-0004.2011.01624.x 21214876

[57] Chou C Soong B Lin K Tsai Y Jih K Liao Y. Clinical characteristics of Taiwanese patients with Hereditary spastic paraplegia type 5. Ann Clin Transl Neurol. (2020) 7:486–96. 10.1002/acn3.51019 32202070 PMC7187706

[58] Fridman V Bundy B Reilly MM Pareyson D Bacon C Burns J. CMT subtypes and disease burden in patients enrolled in the Inherited Neuropathies Consortium natural history study: a cross-sectional analysis. J Neurol Neurosurg Psychiatry. (2015) 86:873–8. 10.1136/jnnp-2014-308826 25430934 PMC4516002

[59] Meyyazhagan A Orlacchio A. Hereditary spastic paraplegia: an update. Int J Mol Sci. (2022) 23:1697. 10.3390/ijms23031697 35163618 PMC8835766

[60] Wang J Fang F Ding C Li J Wu Y Zhang W. Clinical and genetic spectrum of hereditary spastic paraplegia in Chinese children. Dev Med Child Neurol. (2023) 65:416–23. 10.1111/dmcn.15385 36109173

[61] Kara E Tucci A Manzoni C Lynch DS Elpidorou M Bettencourt C. Genetic and phenotypic characterization of complex hereditary spastic paraplegia. Brain. (2016) 139:1904–18. 10.1093/brain/aww111 27217339 PMC4939695

[62] Using Population Descriptors in Genetics and Genomics Research. Washington, DC: National Academies Press (2023). 36989389

[63] Lumaka A Carstens N Devriendt K Krause A Kulohoma B Kumuthini J. Increasing African genomic data generation and sharing to resolve rare and undiagnosed diseases in Africa: a call-to-action by the H3Africa rare diseases working group. Orphanet J Rare Dis. (2022) 17:230. 10.1186/s13023-022-02391-w 35710439 PMC9201791

[64] Manrai AK Funke BH Rehm HL Olesen MS Maron BA Szolovits P. Genetic misdiagnoses and the potential for health disparities. N Engl J Med. (2016) 375:655–65. 10.1056/NEJMsa1507092 27532831 PMC5292722

