# Supplementary material for: The mutational profile in a South African cohort with inherited neuropathies and spastic paraplegia
Source: Front Neurol. 2023 Aug 29;14:1239725. doi: 10.3389/fneur.2023.1239725 (PMC10497947; doi:10.3389/fneur.2023.1239725)
Supplement: Supplementary file 1 [file Table_1.docx]

Supplementary table 1. Summary of the methods used to perform whole exome and whole genome sequencing for the samples in this study.

|  | **Dataset** | **Library kit** | **Sequencing instrument** | **Read length** |
| --- | --- | --- | --- | --- |
| GN and HSP probands | ICGNMD WES | SureSelect All Exon V6 (Agilent) | Illumina NovaSeq 6000 | 2 x 150bp |
|  | UCT-NRG WGS^1,2^ | MGIEasy Universal DNA Library Prep Kit; 30X WGS KAPA PCR-Free v2.1 | BGI MGISEQ-2000; Illumina NovaSeq 6000 | 2 x 150bp |
|  | CReATe WGS^2^ | WGS KAPA HyperPrep | Illumina NovaSeq 6000 | 2 x 150bp |
| controls | UCT-NRG WGS^1,2^ | 30X WGS TruSeq PCR-free | HiSeq X Ten;  Illumina HiSeq 3000 | 2 x 150bp |
|  | SGDP WGS^3^ | unknown | Illumina HiSeq 2000 | 2 x 100bp |
|  | SAHGP WGS^4^ | unknown | Illumina HiSeq 2000 | 2 x 100bp |
|  | AWI-Gen WGS^5^ | unknown | Illumina HiSeq X Ten | 2 x 150bp |
|  | H3Africa Genotyping Chip Project WGS^6^ | TruSeq Nano DNA Library Prep Kit | Illumina HiSeq X Ten | 2 x 150bp |

GN=genetic neuropathy. HSP=hereditary spastic paraplegia. ICGNMD=International Centre for Genomic Medicine in Neuromuscular Diseases. CReATe=Clinical Research in ALS and related disorders for Therapeutic Development Consortium. UCT-NRG=University of Cape Town Neurology Research Group. SGDP=Simons Genome Diversity Project. SAHGP=South African Human Genome Program. AWI-Gen=Africa Wits-INDEPTH Partnership for Genomic Research. H3Africa=The Human Heredity and Health in Africa (H3Africa) consortium.

References

1. Nel M, Mulder N, Europa TA, Heckmann JM. Using Whole Genome Sequencing in an African Subphenotype of Myasthenia Gravis to Generate a Pathogenetic Hypothesis. Front Genet. 2019 Mar 1;10.

2. Nel M, Mahungu AC, Monnakgotla N, Botha GR, Mulder NJ, Wu G, et al. Revealing the Mutational Spectrum in Southern Africans With Amyotrophic Lateral Sclerosis. Neurol Genet. 2022 Feb 12;8(1):e654.

3. Mallick S, Li H, Lipson M, Mathieson I, Gymrek M, Racimo F, et al. The Simons Genome Diversity Project: 300 genomes from 142 diverse populations. Nature. 2016 Oct 21;538(7624):201–6.

4. Choudhury A, Ramsay M, Hazelhurst S, Aron S, Bardien S, Botha G, et al. Whole-genome sequencing for an enhanced understanding of genetic variation among South Africans. Nat Commun. 2017;8(1):1–12.

5. Sengupta D, Choudhury A, Fortes-Lima C, Aron S, Whitelaw G, Bostoen K, et al. Genetic substructure and complex demographic history of South African Bantu speakers. Nat Commun. 2021 Apr 7;12(1):2080.

6. Choudhury A, Aron S, Botigué LR, Sengupta D, Botha G, Bensellak T, et al. High-depth African genomes inform human migration and health. Nature. 2020 Oct 29;586(7831):741–8.
