# Supplementary material for: The mutational profile in a South African cohort with inherited neuropathies and spastic paraplegia
Source: Front Neurol. 2023 Aug 29;14:1239725. doi: 10.3389/fneur.2023.1239725 (PMC10497947; doi:10.3389/fneur.2023.1239725)
Supplement: Supplementary file 2 [file Table_2.docx]

Supplementary table 2. Summary of ACMG criteria codes and their corresponding strength levels applied to the classification of sequence variants according to the ClinGen general sequence variant curation standard operating procedure (version 3.2, October 2022)^7^ and ClinGen sequence variant interpretation working group guidelines^8^.

| **Pathogenic evidence** | | |
| --- | --- | --- |
|  | ***de novo*/dominant** | **recessive** |
| Frequency^8^  **PM2_supporting**  -gnomAD v2.1.1, V3.1.2 subpopulations^a^  -internal control dataset (1074 alleles)^1–6^ | MAF<0.001 | MAF<0.01 |
| Computational prediction (missense variants)^9^  **PP3 met (supporting)**  **PP3_moderate**  **PP3_strong** | REVEL score^10^  0.644-0.773  0.773-0.932  ≥0.932 | |
| Computational prediction (non-canonical splice site variants)^11^  **PP3 met (supporting)** | SpliceAI score^12^  ≥ 0.2 | |
| Predicted null variant^13^  **PVS1_supporting**  **PVS1_moderate**  **PVS1_strong**  **PVS1 met (very strong)** | strength level determined by AutoPVS1^14^ | |
| Segregation^15,b^  **PP1 met (supporting)**  **PP1_moderate**  **PP1_strong** | ≥3 informative meioses across ≥1 family ≥4 informative meioses across ≥1 family ≥5 informative meioses across ≥1 family | ≥1 informative meioses across ≥1 family ≥2 informative meioses across ≥1 family ≥3 informative meioses across ≥1 family |
| Pathogenic missense variant at same amino acid  **PM5 met (moderate)** | award if the missense change has a pathogenic or likely pathogenic classification (without relying on PS1 or PM5) | |
| Functional studies  **PS3_supporting** | functional studies provide supportive evidence that the variant has a damaging effect on the gene or gene product | |
| Mutational hotspot  **PM1 met (supporting)** | variant clusters with other reported pathogenic variants in a specific region of the protein | |
| Observation in other probands^c^  **PS4_supporting**  **PS4_moderate**  **PS4 met (strong)** | ≥ 1 unrelated proband(s) with consistent phenotype for disorder  ≥ 5 unrelated probands with consistent phenotype for disorder  ≥ 10 unrelated probands with consistent phenotype for disorder | |
| Detected in *trans* with pathogenic variant^d^  **PM3_supporting**  **PM3 met (moderate)**  **PM3_strong**  **PM3_verystrong** |  | 0.5 points  1.0 point  2.0 points  4.0 points |
| *De novo* observations^e^  **PS2_supporting or PM6_supporting**  **PS2_moderate or PM6 met (moderate)**  **PS2 met (strong) or PM6_strong**  **PS2_verystrong or PM6_verystrong** | 0.5 points  1.0 point  2 points  4 points |  |
| **Benign evidence** | | |
| Computational prediction (missense variants^9^  **BP4 met (supporting)**  **BP4_moderate**  **BP4_strong**  **BP4_verystrong** | REVEL score^10^  0.183-0.290  0.016-0.183  0.003-0.016  ≤0.003 | |
| Computational prediction (non-canonical splice site variants)^11^  **BP4 met (supporting)** | SpliceAI score^12^  ≥ 0.1 | |
| Non-segregation  **BS4_supporting**  **BS4 met (strong)** | variant does not segregate with 1 informative meiosis in 1 family  variant does not segregate with ≥ 2 informative meioses across ≥ 1 family | |
| **BS2 not applied** | NMD phenotypes complicated by late onset and reduced penetrance of pathogenic variants | |

a. gnomAD coverage at the variant site must be ≥ 20X. b. Obligate carriers may be counted; unaffected individuals are not counted; monozygotic twins are counted as 1 segregation. c. Exclude compound heterozygous proband observations (scored towards PM3) from proband counting towards PS4.

d. Recommended point-based scoring of variants for PM3 criterion^7^.

| **Classification/zygosity of other variant** | **Points per proband** | |
| --- | --- | --- |
|  | **Confirmed in *trans*** | **Phase unknown** |
| Pathogenic (P) or likely pathogenic (LP) variant | 1.0 | 0.5 (P)  0.25 (LP) |
| Homozygous occurrence (max point 1.0) | 0.5 | NA |
| Uncertain significance variant (max point 0.5) | 0.25 | 0 |

NA=not applicable.

e. Recommended point-based scoring of variant *de novo* observations^7^.

| **Phenotypic consistency** | **Points per proband** | |
| --- | --- | --- |
|  | **Confirmed *de novo*** | **Assumed *de novo*** |
| Phenotype highly specific for gene | 2 | 1 |
| Phenotype consistent with gene but not highly specific | 1 | 0.5 |
| Phenotype consistent with gene but not highly specific and high genetic heterogeneity* | 0.5 | 0.25 |
| Phenotype consistent with gene | 0 | 0 |

* maximum allowable value of 1 may contribute to overall score.

References

1. Nel M, Mulder N, Europa TA, Heckmann JM. Using Whole Genome Sequencing in an African Subphenotype of Myasthenia Gravis to Generate a Pathogenetic Hypothesis. Front Genet. 2019 Mar 1;10.

2. Nel M, Mahungu AC, Monnakgotla N, Botha GR, Mulder NJ, Wu G, et al. Revealing the Mutational Spectrum in Southern Africans With Amyotrophic Lateral Sclerosis. Neurol Genet. 2022 Feb 12;8(1):e654.

3. Mallick S, Li H, Lipson M, Mathieson I, Gymrek M, Racimo F, et al. The Simons Genome Diversity Project: 300 genomes from 142 diverse populations. Nature. 2016 Oct 21;538(7624):201–6.

4. Choudhury A, Ramsay M, Hazelhurst S, Aron S, Bardien S, Botha G, et al. Whole-genome sequencing for an enhanced understanding of genetic variation among South Africans. Nat Commun. 2017;8(1):1–12.

5. Sengupta D, Choudhury A, Fortes-Lima C, Aron S, Whitelaw G, Bostoen K, et al. Genetic substructure and complex demographic history of South African Bantu speakers. Nat Commun. 2021 Apr 7;12(1):2080.

6. Choudhury A, Aron S, Botigué LR, Sengupta D, Botha G, Bensellak T, et al. High-depth African genomes inform human migration and health. Nature. 2020 Oct 29;586(7831):741–8.

7. The Clinical Genome Resource ClinGen Variant Curation SOP Committee. ClinGen General Sequence Variant Curation Process Standard Operating Procedure.

8. Harrison SM, Biesecker LG, Rehm HL. Overview of Specifications to the ACMG/AMP Variant Interpretation Guidelines. Curr Protoc Hum Genet. 2019;103(1):e93.

9. Pejaver V, Byrne AB, Feng B, Pagel KA, Mooney SD, Karchin R, et al. Calibration of computational tools for missense variant pathogenicity classification and ClinGen recommendations for PP3/BP4 criteria. Am J Hum Genet. 2022 Dec;109(12):2163–77.

10. Ioannidis NM, Rothstein JH, Pejaver V, Middha S, McDonnell SK, Baheti S, et al. REVEL: An Ensemble Method for Predicting the Pathogenicity of Rare Missense Variants. Am J Hum Genet. 2016;99(4):877–85.

11. Walker LC, Hoya M de la, Wiggins GAR, Lindy A, Vincent LM, Parsons MT, et al. Application of the ACMG/AMP framework to capture evidence relevant to predicted and observed impact on splicing: recommendations from the ClinGen SVI Splicing Subgroup. medRxiv. 2023 Jan 1;

12. Jaganathan K, Kyriazopoulou Panagiotopoulou S, McRae JF, Darbandi SF, Knowles D, Li YI, et al. Predicting Splicing from Primary Sequence with Deep Learning. Cell. 2019;176(3):535-548.e24.

13. Abou Tayoun AN, Pesaran T, DiStefano MT, Oza A, Rehm HL, Biesecker LG, et al. Recommendations for interpreting the loss of function PVS1 ACMG/AMP variant criterion. Hum Mutat. 2018;39(11):1517–24.

14. Xiang J, Peng J, Baxter S, Peng Z. AutoPVS1: An automatic classification tool for PVS1 interpretation of null variants. Hum Mutat. 2020 Sep 29;41(9):1488–98.

15. Jarvik GP, Browning BL. Consideration of Cosegregation in the Pathogenicity Classification of Genomic Variants. Am J Hum Genet. 2016 Jun 2;98(6):1077–81.
