# Supplementary material for: The mutational profile in a South African cohort with inherited neuropathies and spastic paraplegia
Source: Front Neurol. 2023 Aug 29;14:1239725. doi: 10.3389/fneur.2023.1239725 (PMC10497947; doi:10.3389/fneur.2023.1239725)
Supplement: Supplementary file 3 [file Table_3.docx]

Supplementary table 3. Clinical and electrophysiological characteristics of axonal hereditary sensori-motor neuropathy (HSMN)/CMT2 patients with p.Gln36Ter and p.Arg125Trp *MPV17* variants.

| **Individual** | **Ancestry** | **AAO (years)** | **Age at f-up (years)** | **Phenotype features plus CMT2** | **G&S Sensory modality dysfunction** | **Deep tendon reflexes** | **Electrophysiology detail** | |
| --- | --- | --- | --- | --- | --- | --- | --- | --- |
|  |  |  |  |  |  |  | **CMAP** | **SNAP** |
| fam_007# | SAB (isiXhosa) | 5 | 16 | proximal, diaphragmatic & distal weakness; foot drop + laryngeal palsies; hammer toes | Pin, temp, vibration, proprioception | Arms N  Legs areflexia | Median N  Tibial NR | Median, Ulnar N  Sural NR |
| ICGNMD_7 | SAB (isiXhosa) | 13 | 30 | dysesthesia; weak intrinsics (FDI 0/5, APB 1-2, finger ext 3); floppy feet | mainly pin, temp >> proprioception | Areflexia | Tibial NR  Median  bilat prolonged DLs, CMAPs reduced | Bilat ulnar NR, R median NR, L sural NR  L median reduced SNAP |
| ICGNMD_9 | SAB (isiXhosa) | 20 | 49 | pes planus; exercise-cramps, foot drop | pin, temp, vibration, proprioception | Areflexia with reduced triceps | Median N  Tibial NR | Median, Ulnar N  Sural NR |

AAO refers to age at symptom onset; age at last f-up (follow-up) in years to indicate the confidence that the case remained solely with neuropathy and did not develop a multi-system disease such as hearing loss, myopathy, scoliosis, ptosis. SAB=South African Black. *First dorsal interosseus (FDI) muscle MRC grade 0/5; Abductor pollicis brevis (APB) grade 3/5 and finger extensors 4+/5; foot drop refers to ankle dorsiflexors 0/5 compared to plantar flexors ≥ 2/5; floppy feet refers to ankle dorsiflexors and plantar flexors, foot inverters and evertors all with MRC grade 0/5. NR – no response; N – normal; ND – not done. G&S refers to glove and stocking. Temp refers to temperature sensibility. CMT2 refers to evidence of axonal sensori-motor neuropathy. #HIV-infected vertical transmission– well controlled on antiretroviral therapy. Electrophyiology: CMAP compound motor action potential, SNAP sensory nerve action potential; DL distal latency, bilat bilateral. Due to the propensity of ulnar nerve and common peroneal nerves to entrapment we only report on median and tibial motor nerves.
