# Supplementary material for: The mutational profile in a South African cohort with inherited neuropathies and spastic paraplegia
Source: Front Neurol. 2023 Aug 29;14:1239725. doi: 10.3389/fneur.2023.1239725 (PMC10497947; doi:10.3389/fneur.2023.1239725)

Supplementary figure 1. Integrative Genomics Viewer (IGV) screenshots and Sanger sequencing chromatograms for GN and HSP variants reported in manuscript tables 2 and 3.

A. fam_006 GRCh38 g.11998786 A>G (het) *MFN2* NP_055689.1:p.Thr206Ala

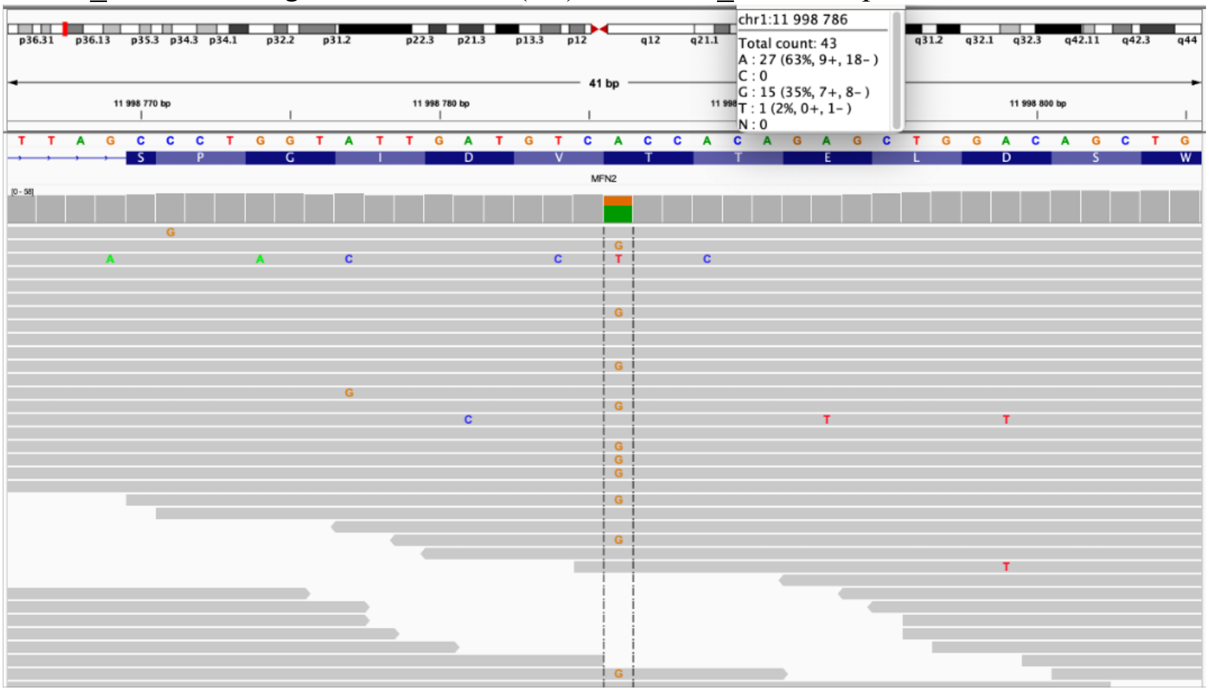

B. fam_081 GRCh38 g.11999054 C>T (het) *MFN2* NP_055689.1:p.Arg259Cys

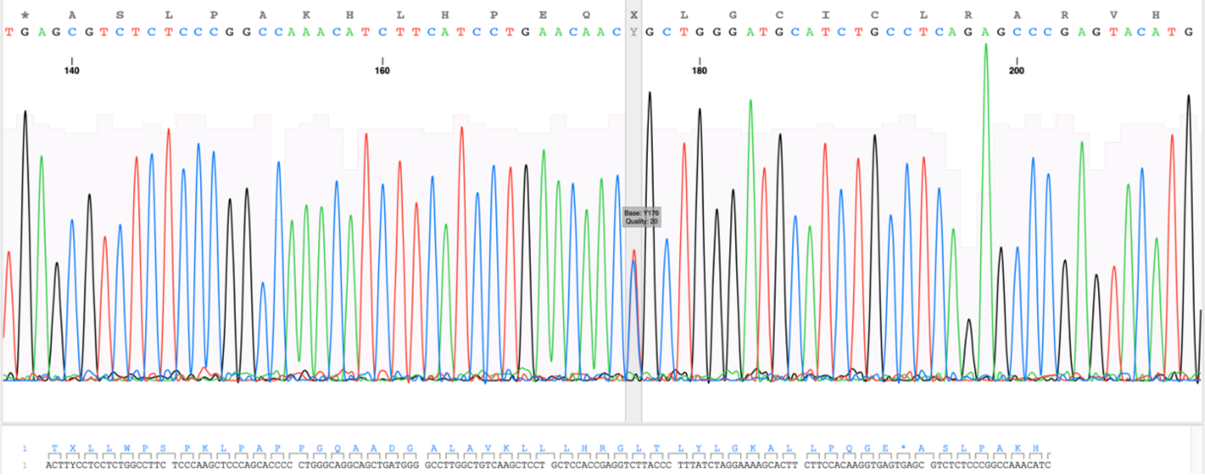

C. fam_083 GRCh38 g.12001423 G>A (het) *MFN2* NP_055689.1:p.Arg280His

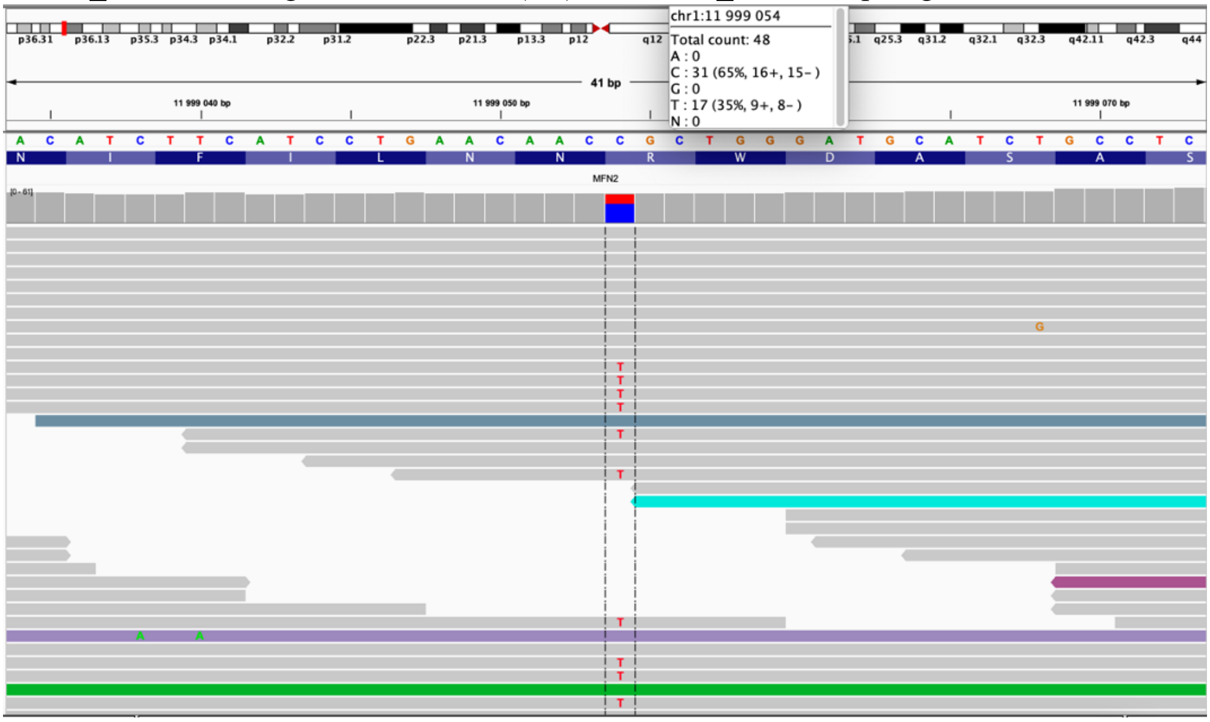

D. ICGNMD_18 GRCh38 g.30941503 G>A (het) *MORC2* NP_001290185.1:p.Arg252Trp

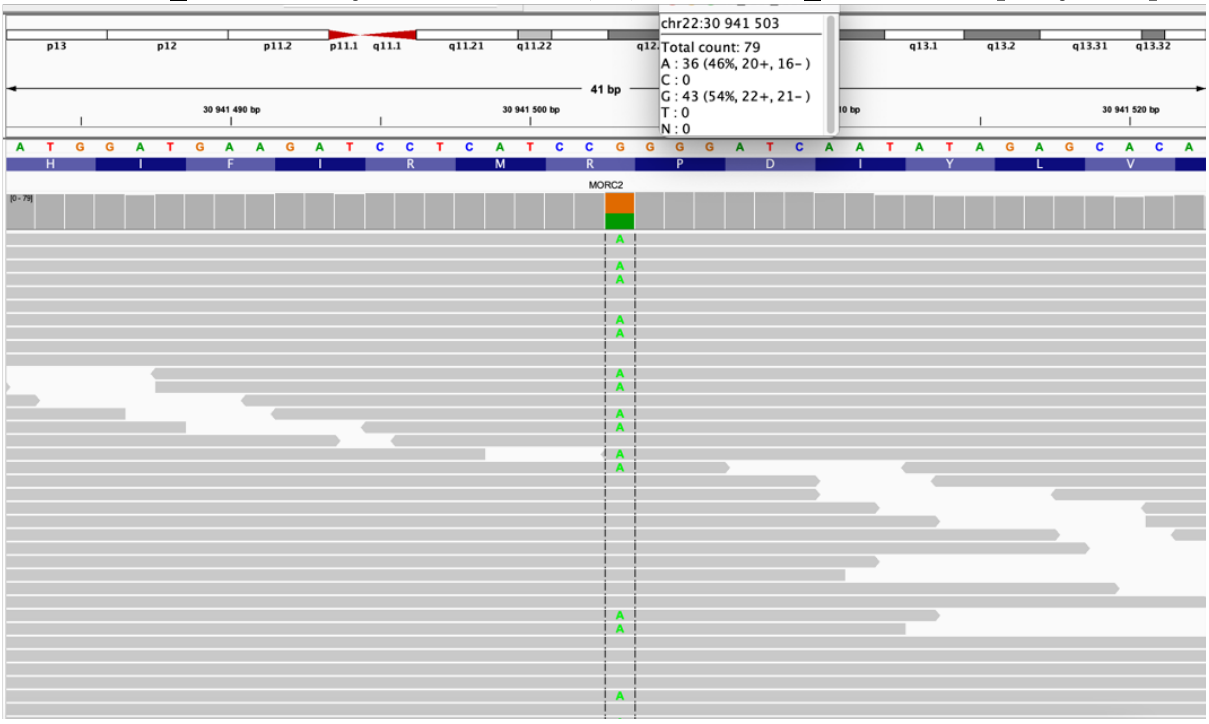

E. ICGNMD_16 GRCh38 g.116395224 T>C (het) *ATP1A1* NP_000692.2:p.Ile592Thr

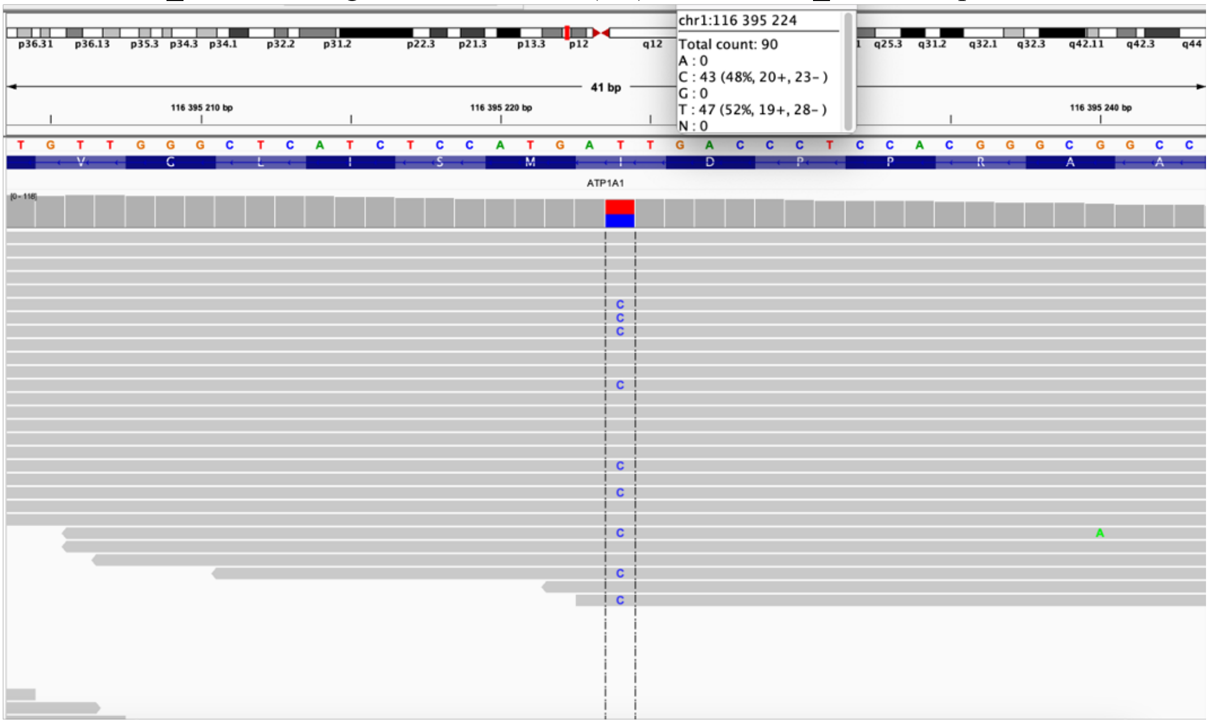

F. ICGNMD_59 GRCh38 g.71223771 C>T (het) *GJB1* NP_000157.1:p.Arg22Ter

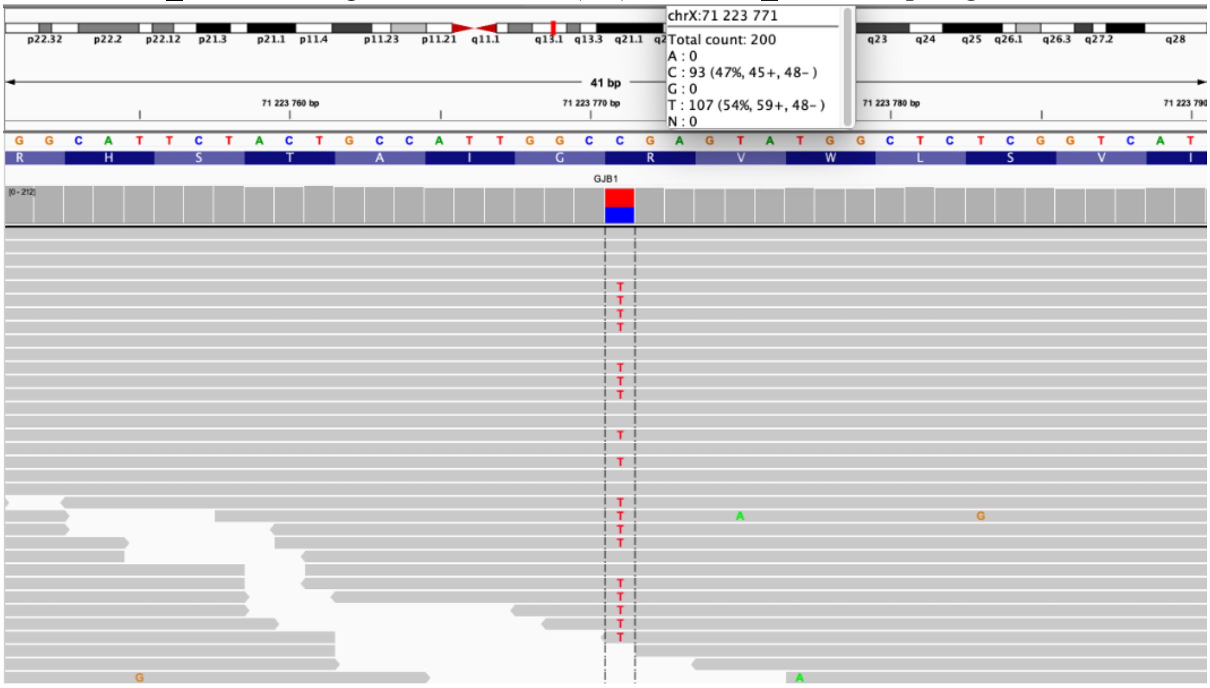

G. ICGNMD_17 GRCh38 g.36093298 T>G (hom) *ADPRS* NP_060295.1:p.Val335Gly

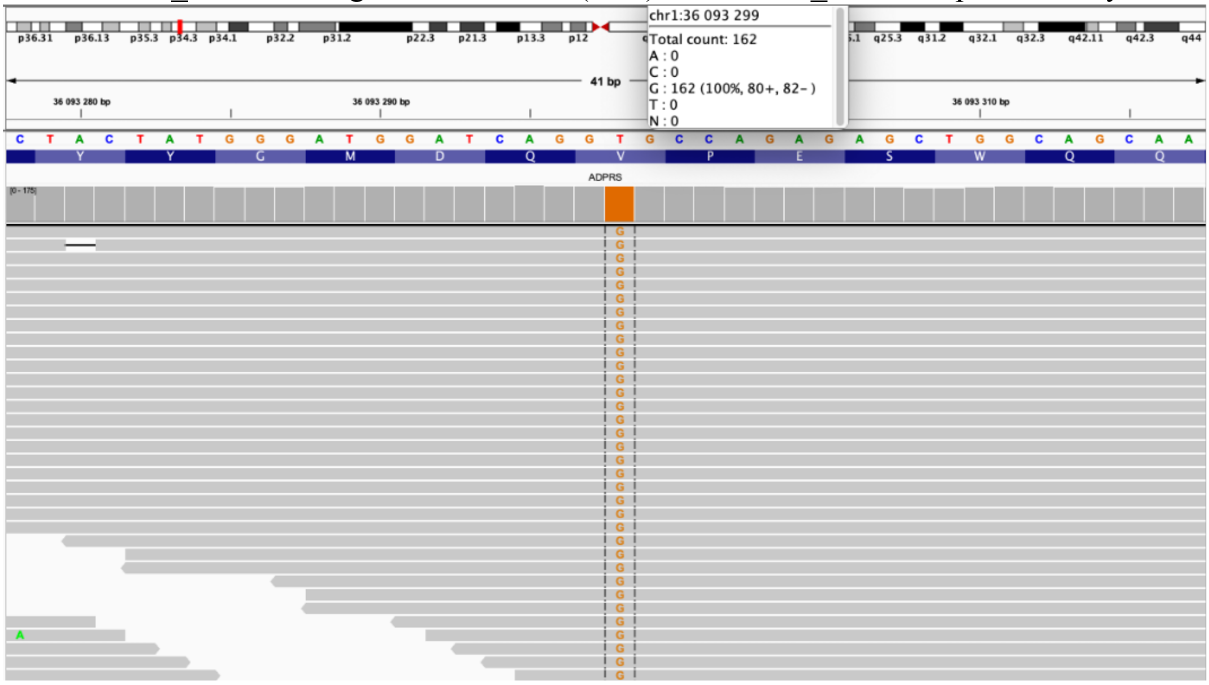

H. ICGNMD_4 GRCh38 g.108301698 C>T (hom) *ATM* NP_000042.3:p.Thr1743IIe

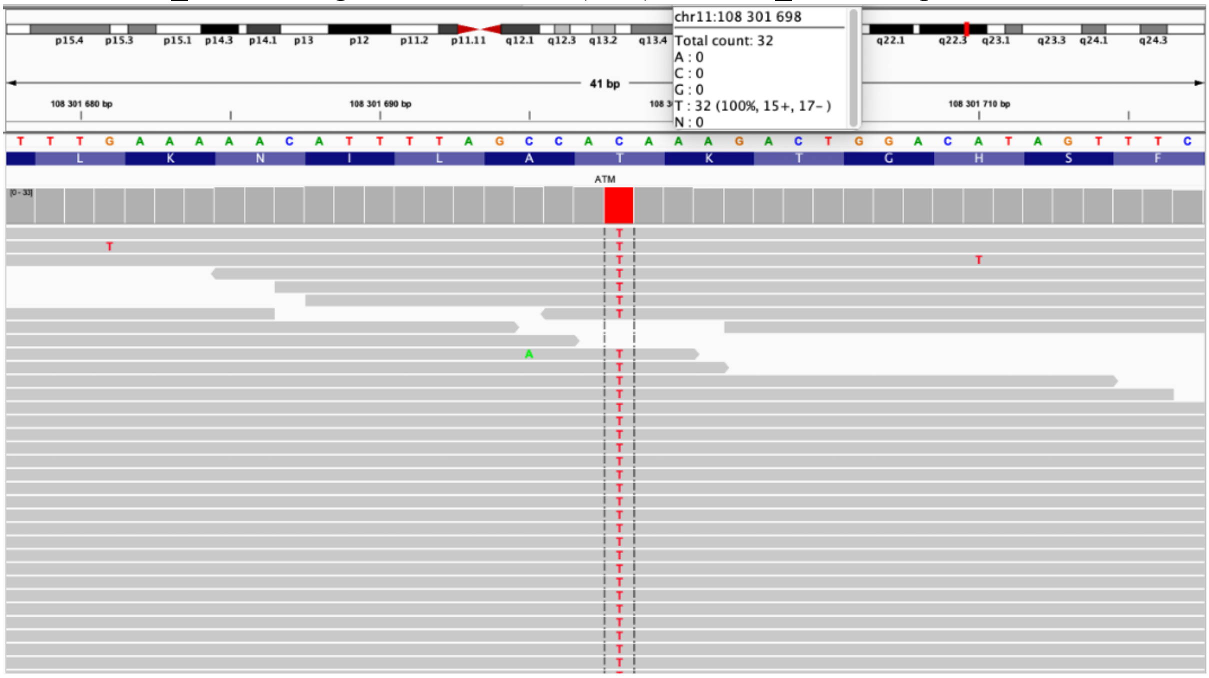

I. i. ICGNMD_15 GRCh38 g.81351695 C>T (het) *GAN* NP_071324.1:p.Gln94Ter

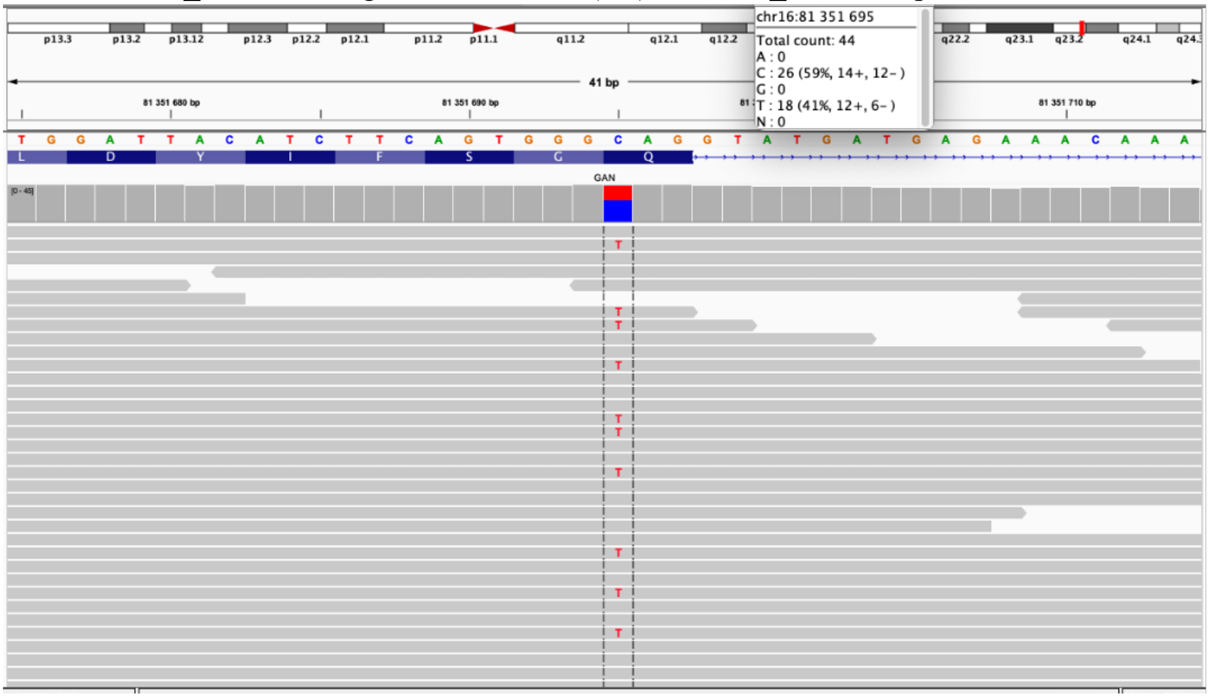

ii. ICGNMD_15 GRCh38 g.81357902 C>T (het) *GAN* NP_071324.1:p.Pro315Leu

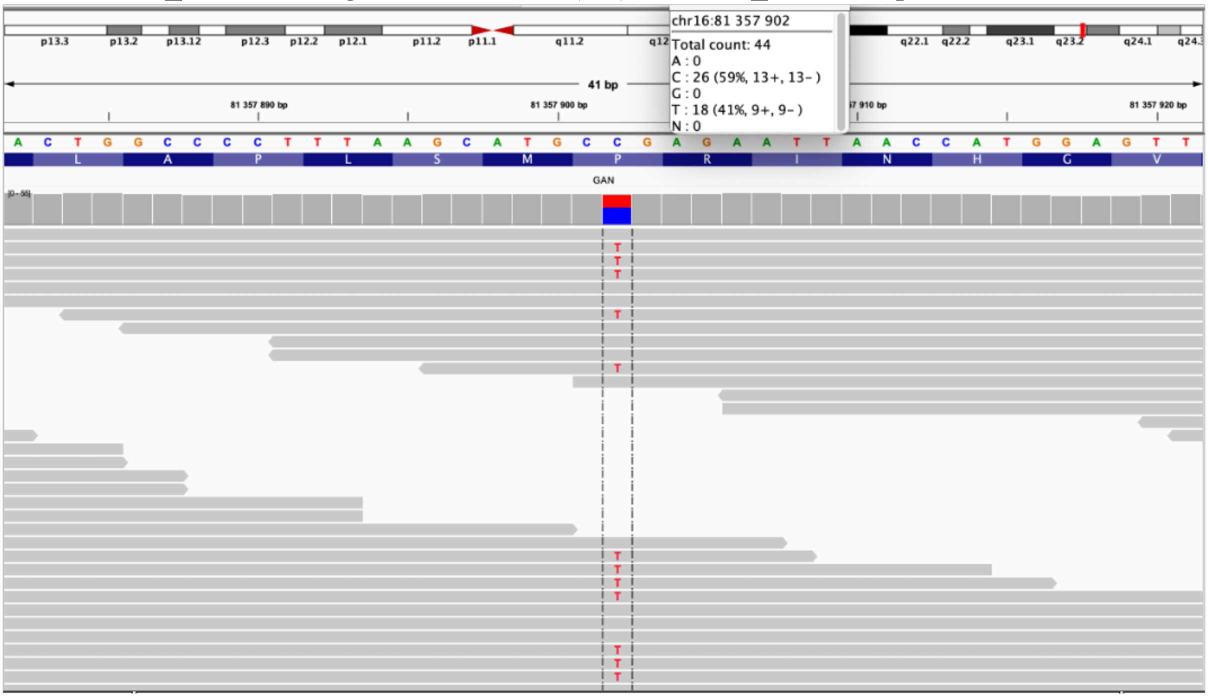

J. i. ICGNMD_6 GRCh38 g.161307280 T>C (het) *MPZ* NP_000521.2:p.Glu71Gly

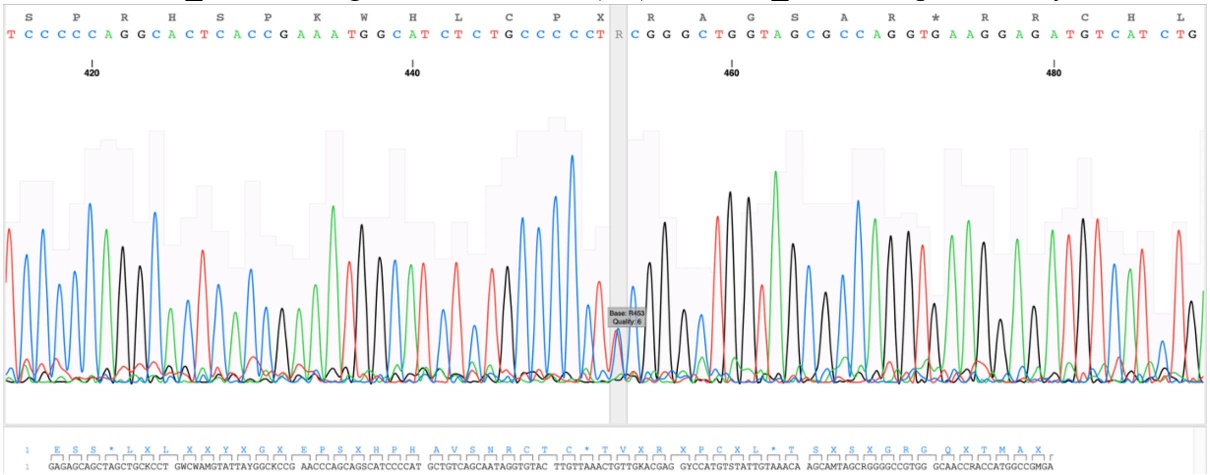

ii. Affected mother of ICGNMD_6 GRCh38 g.161307280 T>C (het) *MPZ* NP_000521.2:p.Glu71Gly

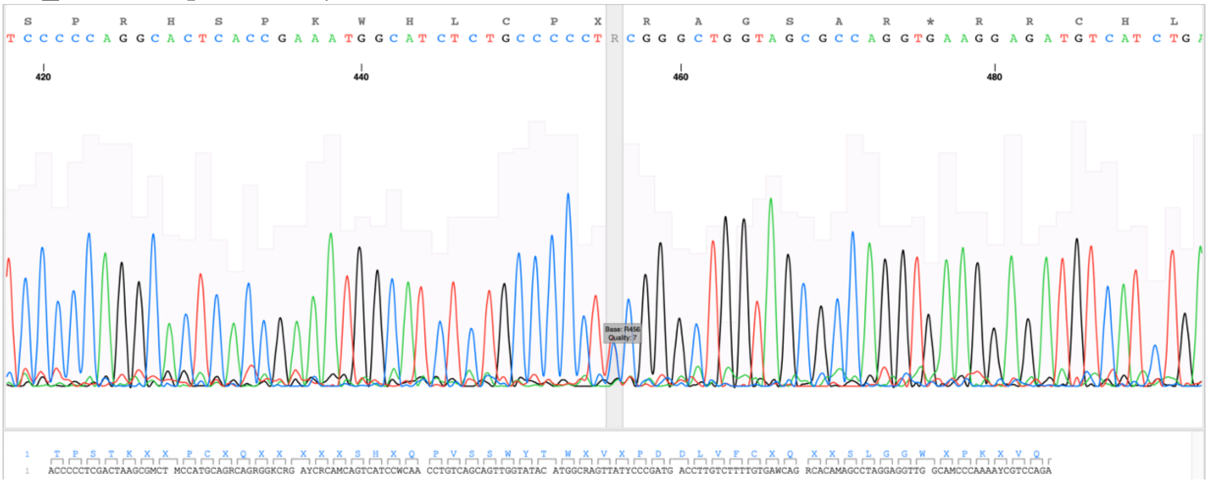

iii. Affected brother of ICGNMD_6 GRCh38 g.161307280 T>C (het) *MPZ* NP_000521.2:p.Glu71Gly

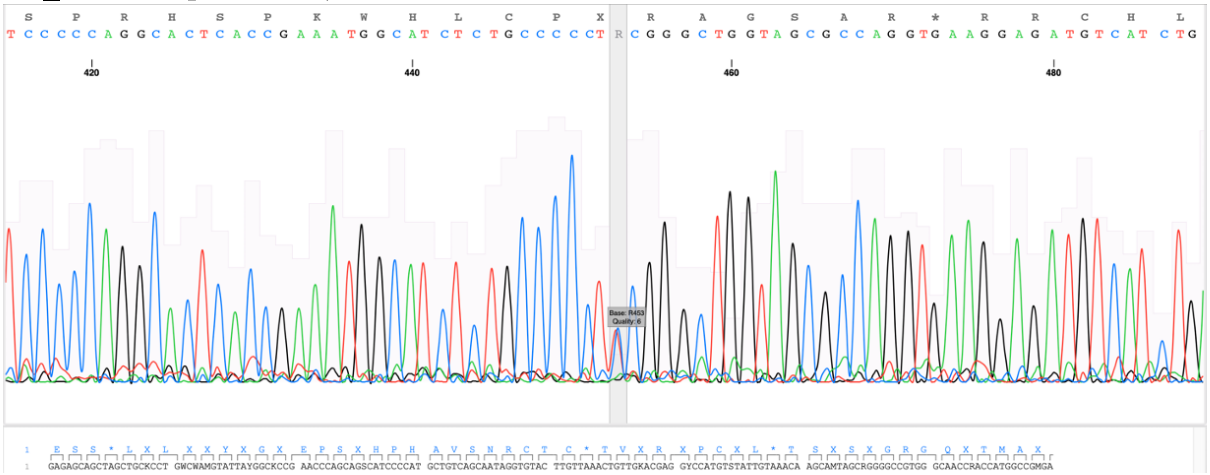

K. i. fam_007 GRCh38 g.27313074 G>A (het) *MPV17* NP_002428.1:p.Gln36Ter

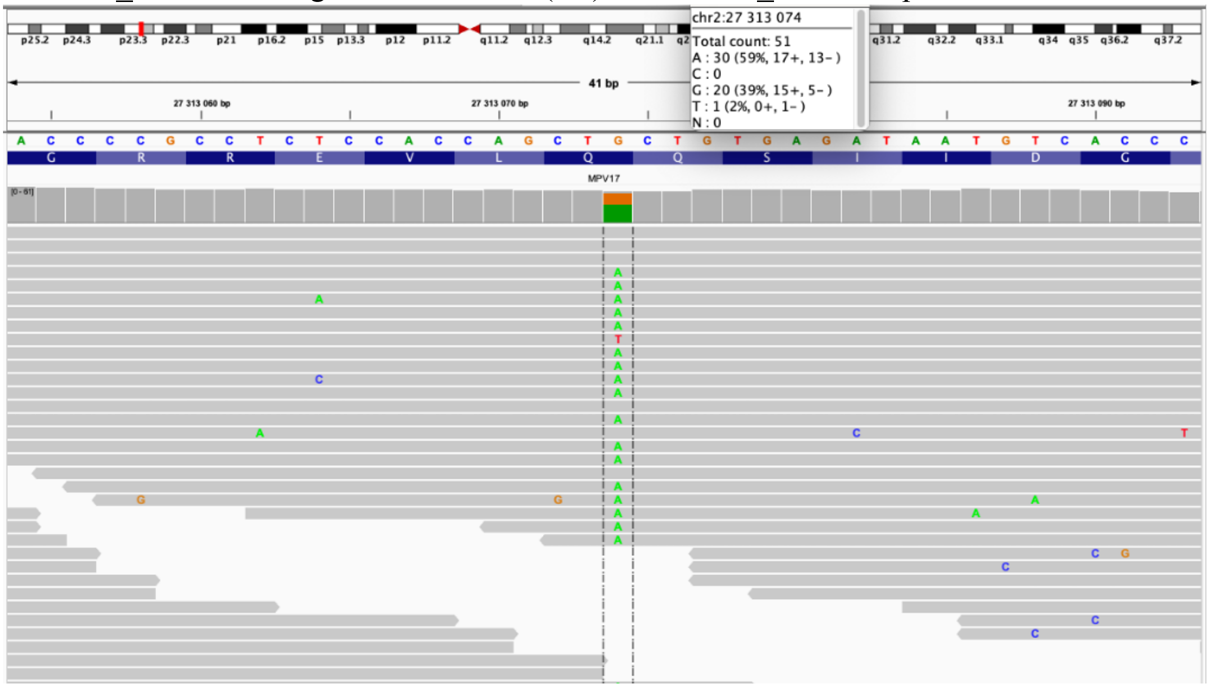

ii. ICGNMD_7 GRCh38 g.27313074 G>A (het) *MPV17* NP_002428.1:p.Gln36Ter

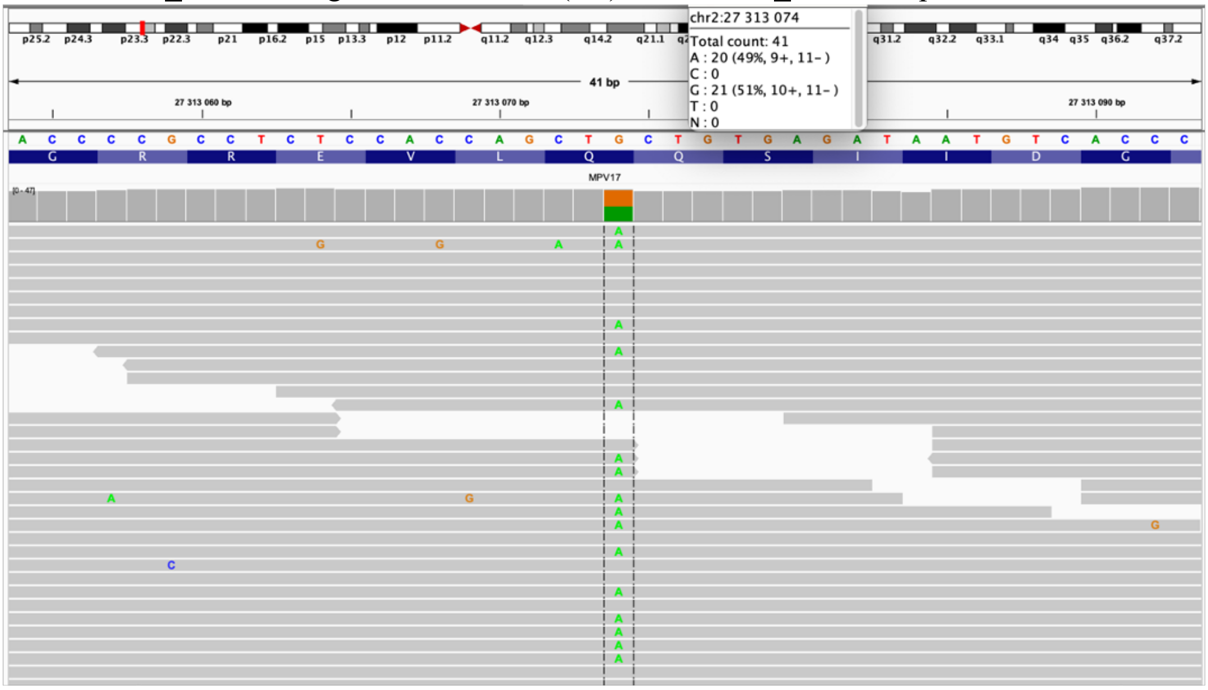

iii. ICGNMD_9 GRCh38 g.27313074 G>A (het) *MPV17* NP_002428.1:p.Gln36Ter

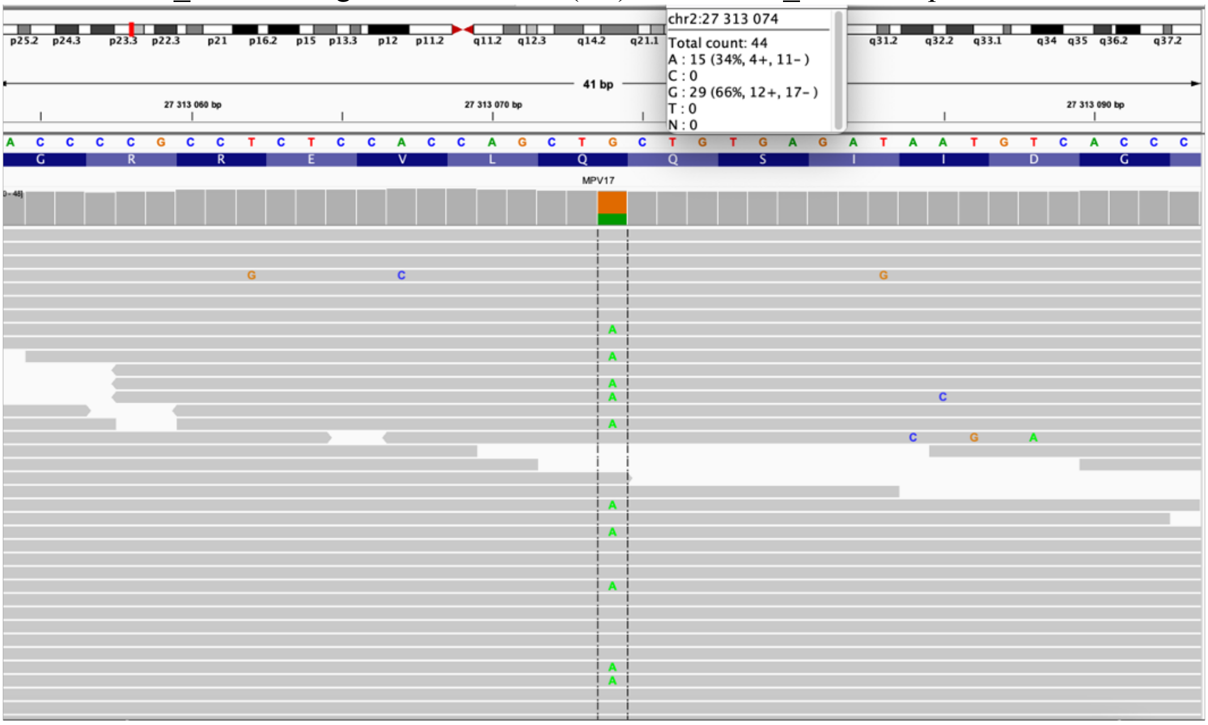

L. i. fam_007 GRCh38 g.27312496 G>A (het) *MPV17* NP_002428.1:p.Arg125Trp

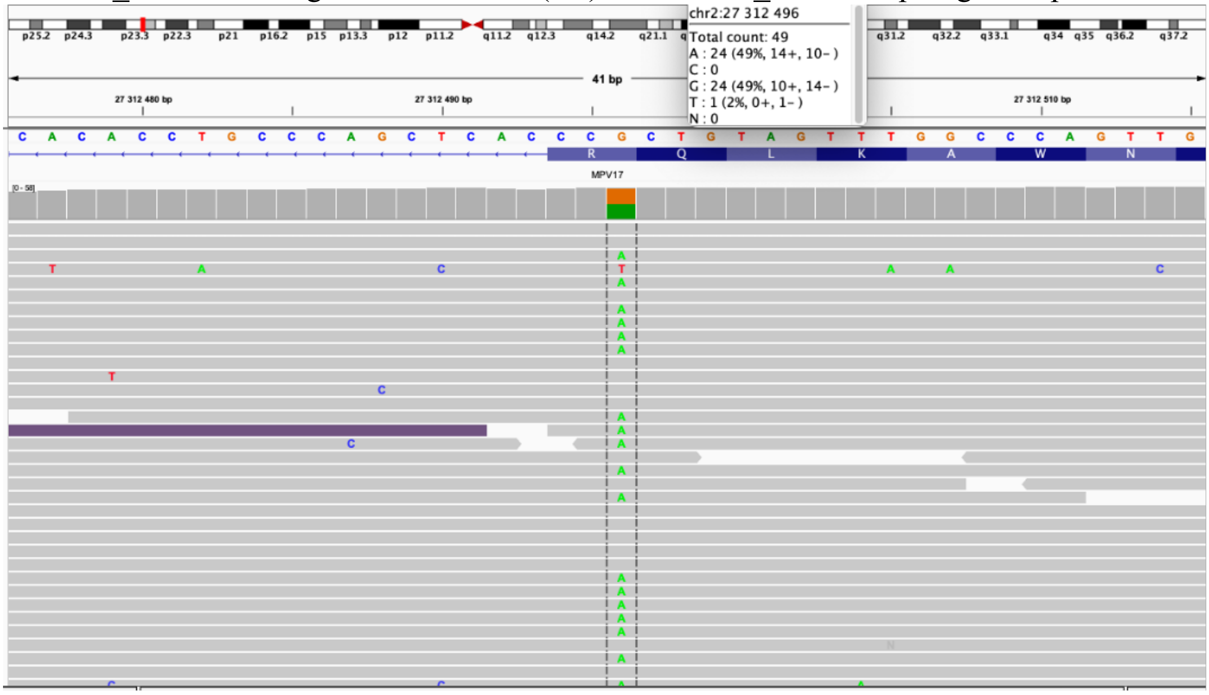

ii. ICGNMD_7 GRCh38 g.27312496 G>A (het) *MPV17* NP_002428.1:p.Arg125Trp

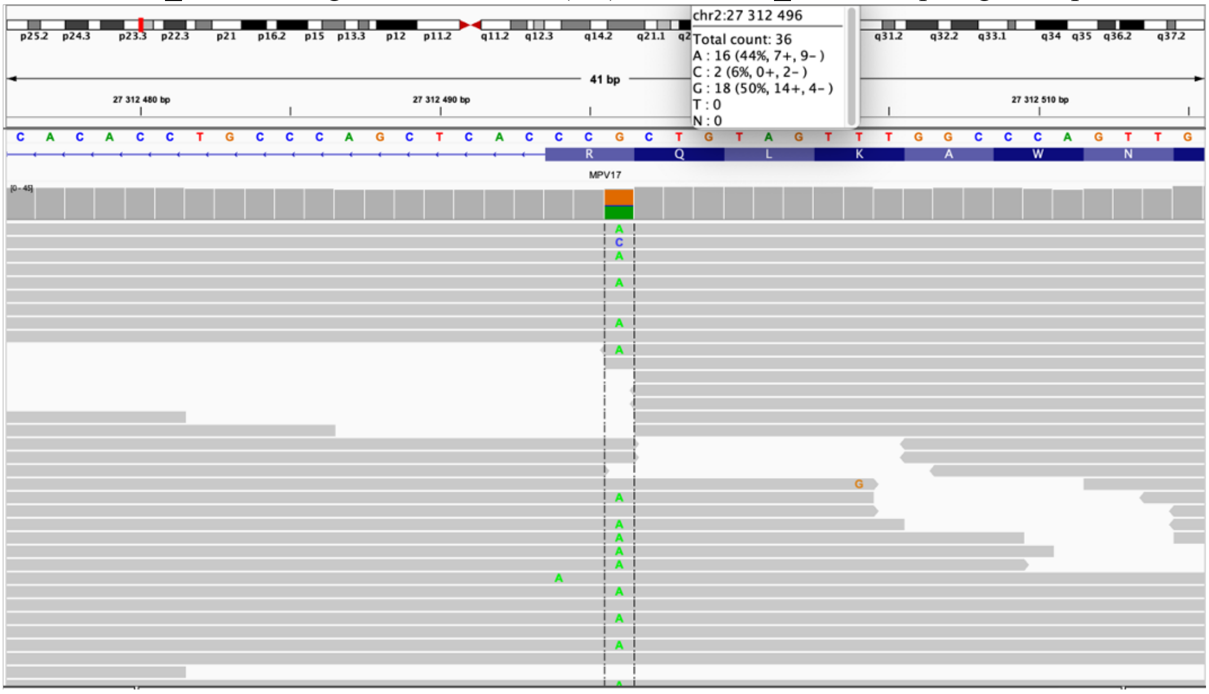

iii. ICGNMD_9 GRCh38 g.27312496 G>A (het) *MPV17* NP_002428.1:p.Arg125Trp

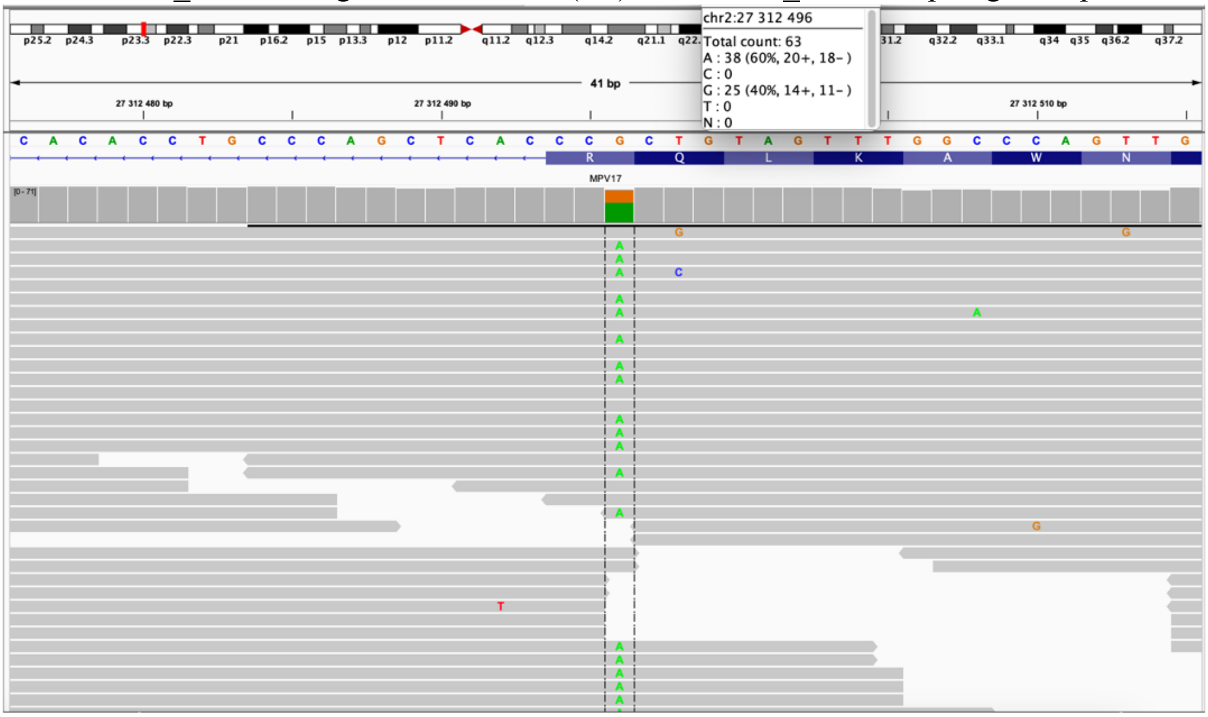

M. i. fam_001 GRCh38 g.240783773 G>T (het) *KIF1A* NP_001230937.1:p.Ala255Asp

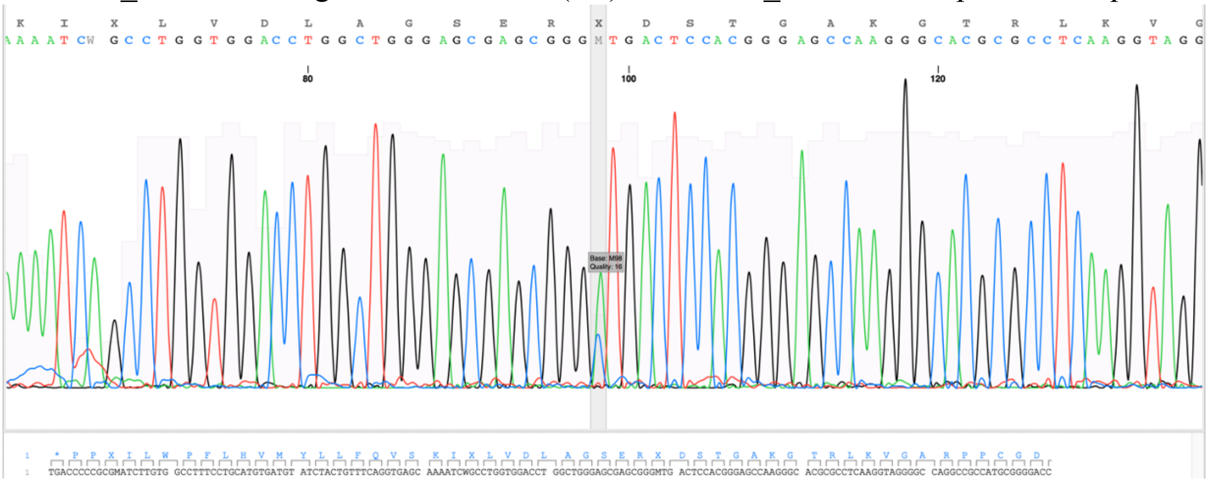

ii. Affected daughter of fam_001 GRCh38 g.240783773 G>T (het) *KIF1A* NP_001230937.1:p.Ala255Asp

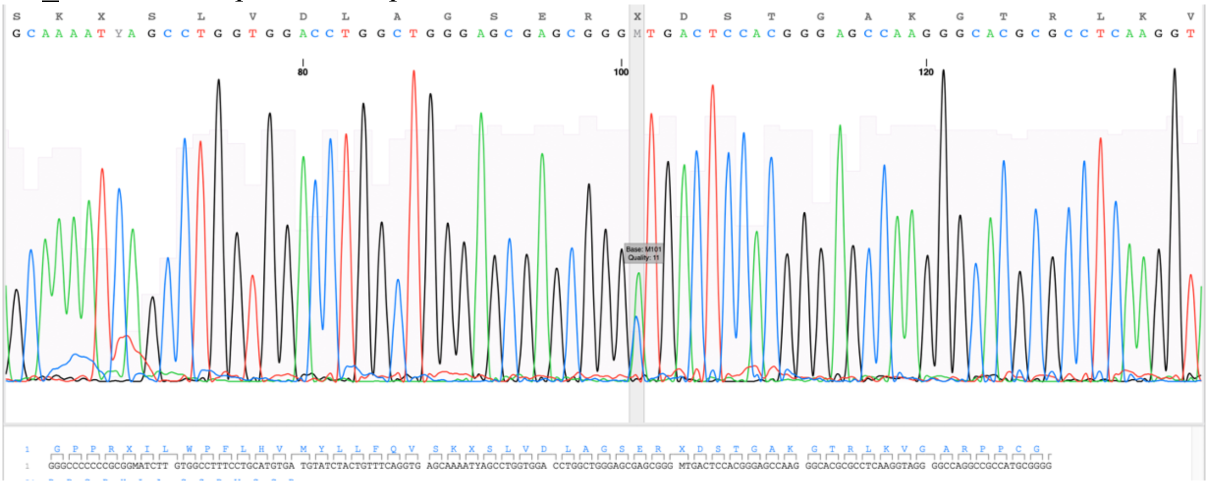

N. ICGNMD_62 GRCh38 g.240774199 T>G (het) *KIF1A* NP_001230937.1:p.Thr341Pro

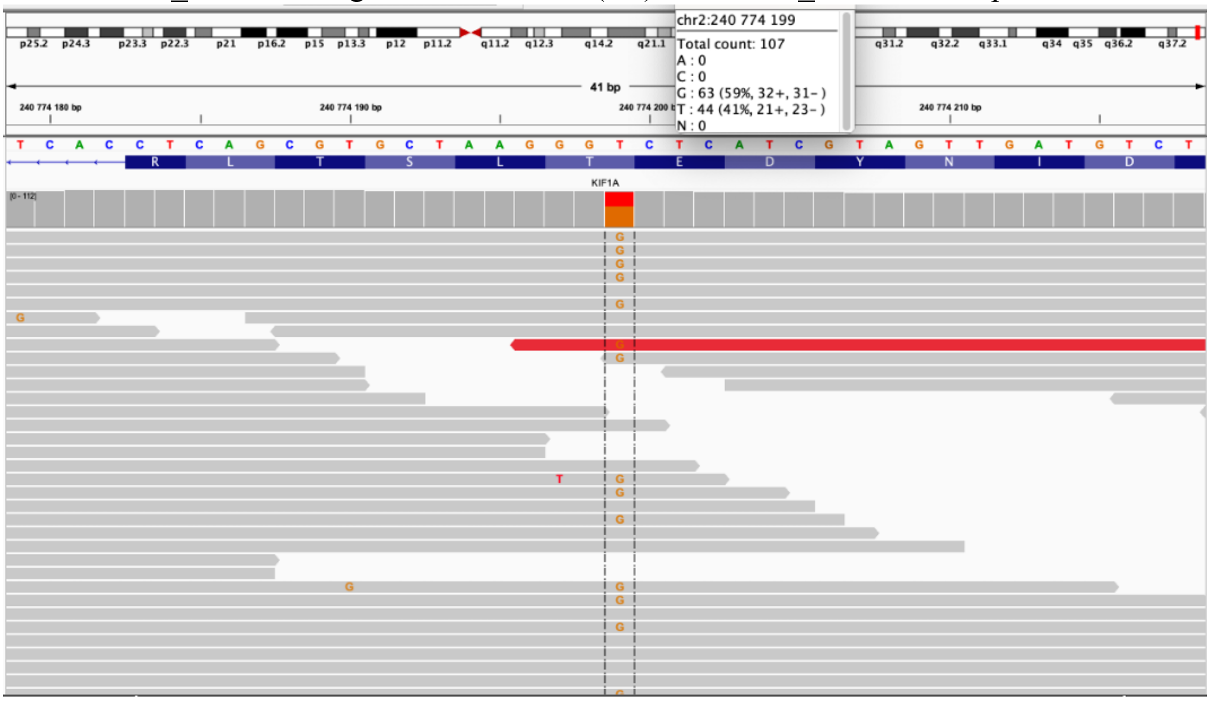

O. fam_059 GRCh38 g.81906541 C>T (hom) *PCYT2* NP_002852.1:p.Gly228Arg

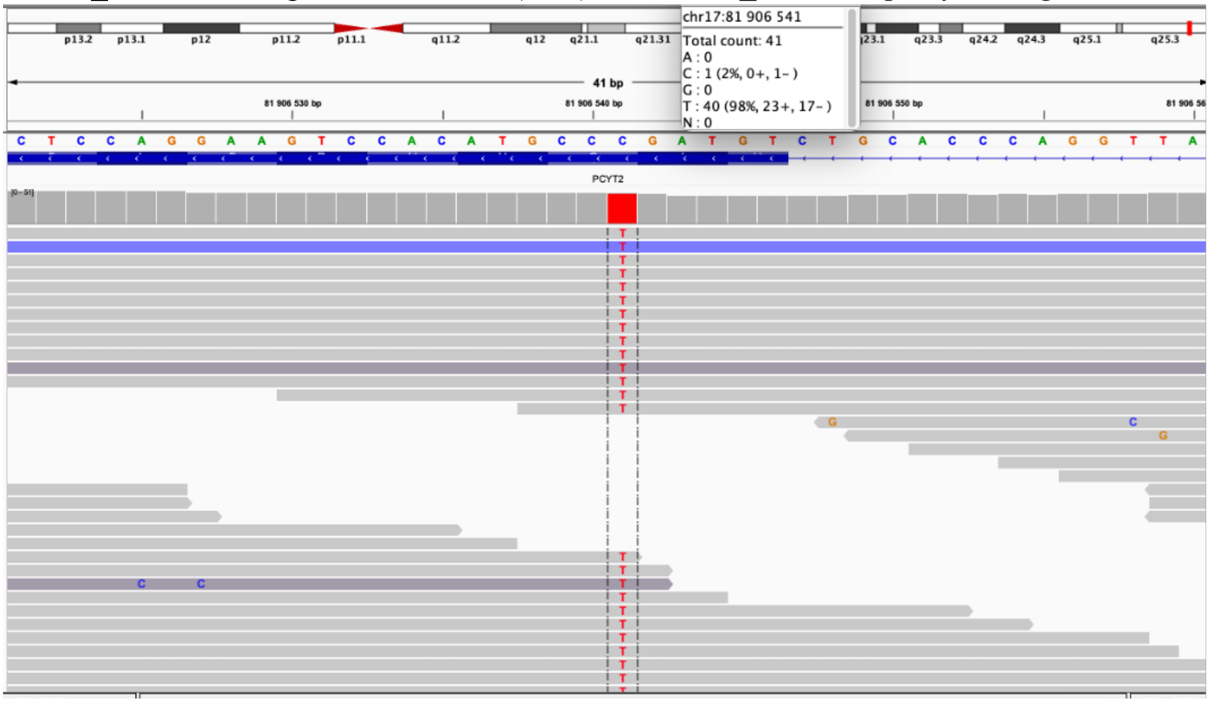

P. fam_111 GRCh38 g.73198093 A>G (het) *PSEN1* NP_000012.1:p.Arg278Gly

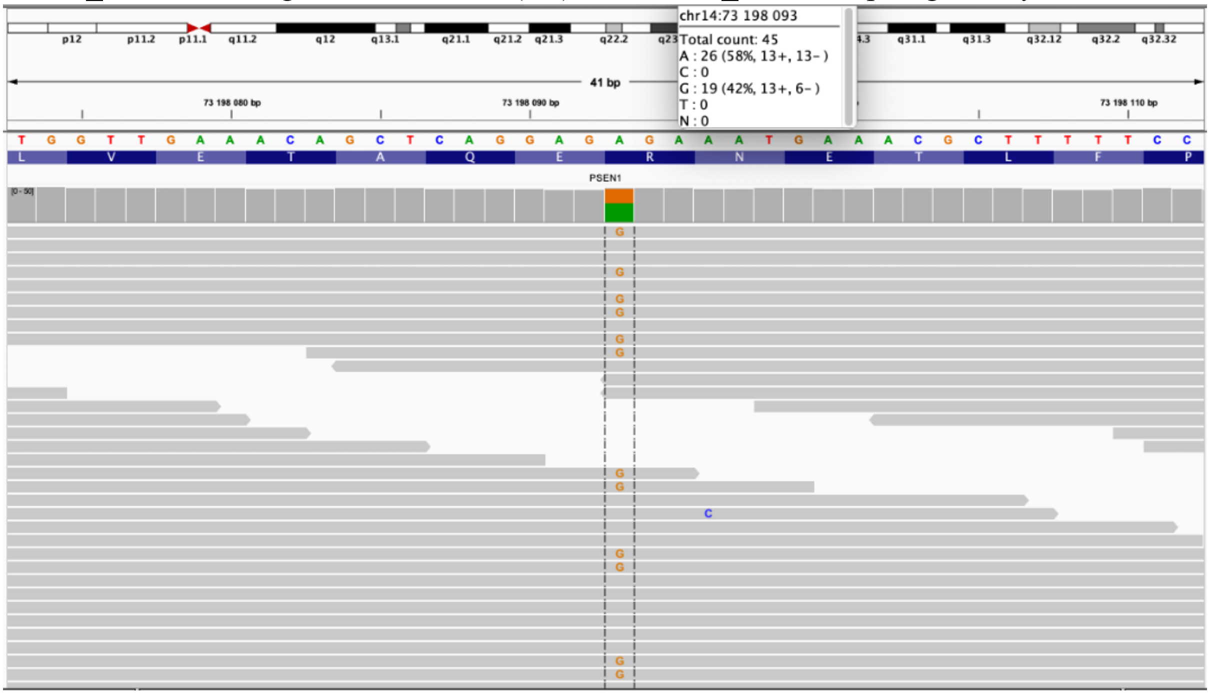

Q. ICGNMD_29 GRCh38 g.50628119 G>C (het) *ATL1* NP_056999.2:p.Arg403Pro

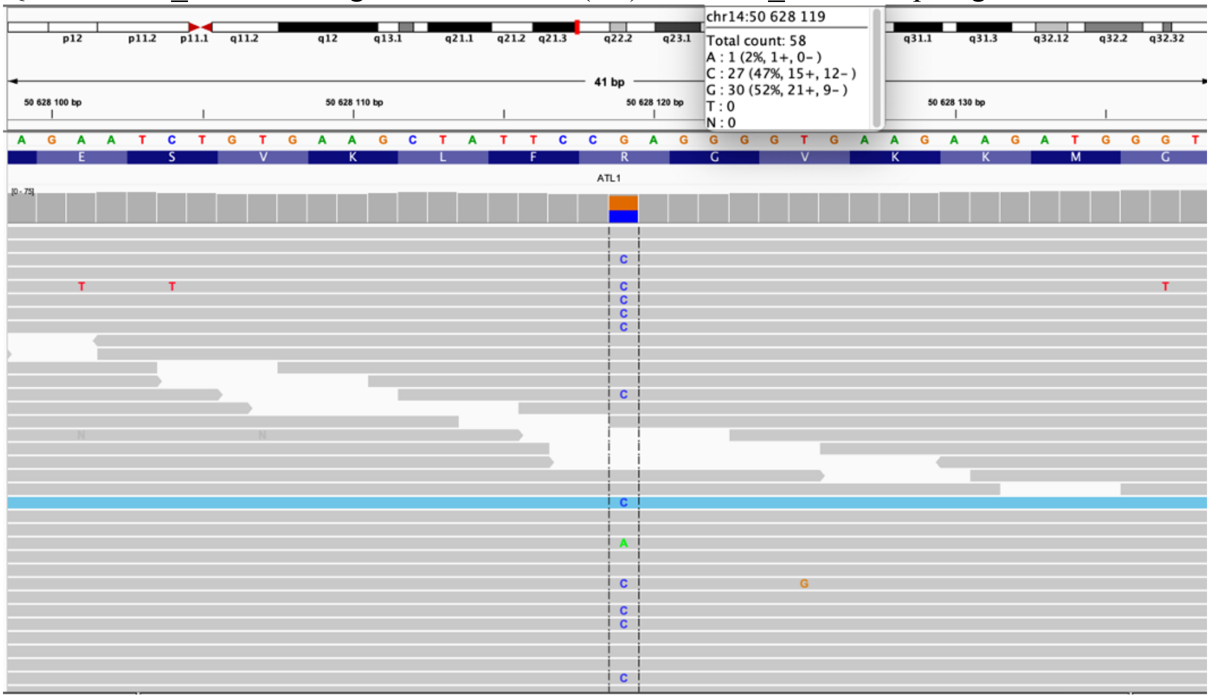

R. ICGNMD_49 GRCh38 g.89546737 C>T (hom) *SPG7* NP_003110.1:p.Ala510Val

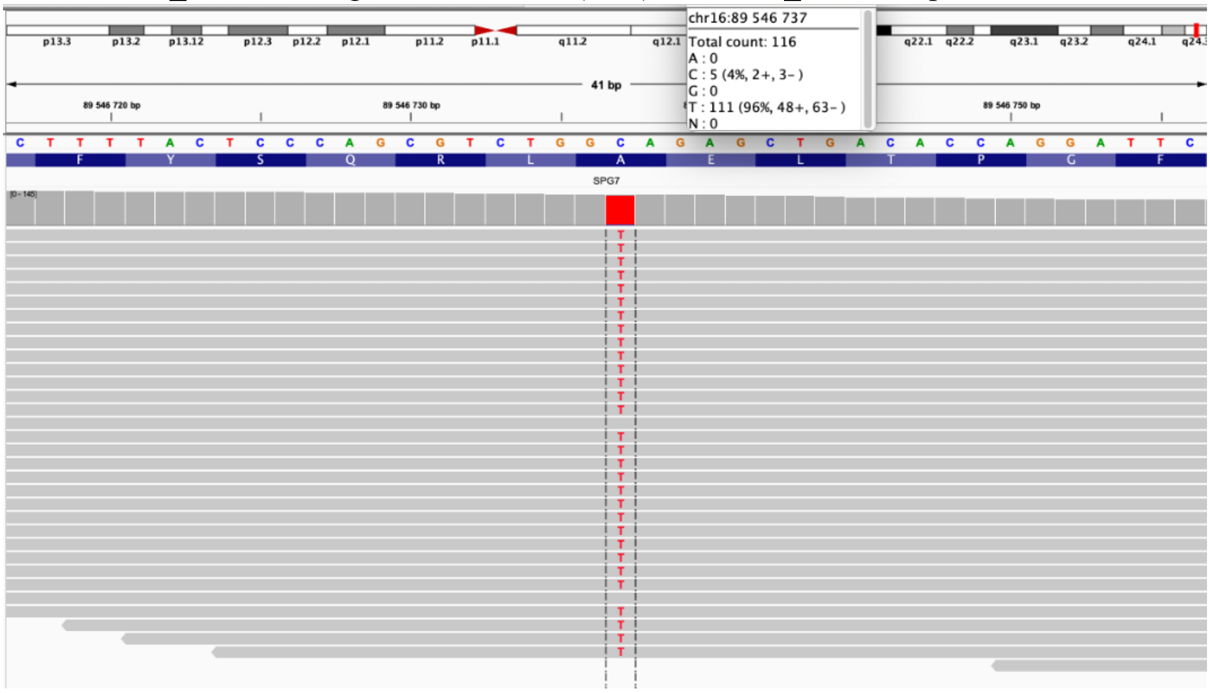

S. i. ICGNMD_22 GRCh38 g.44574917CAGA (het) *SPG11*
NP_079413.3:p.Leu1997MetfsTer60

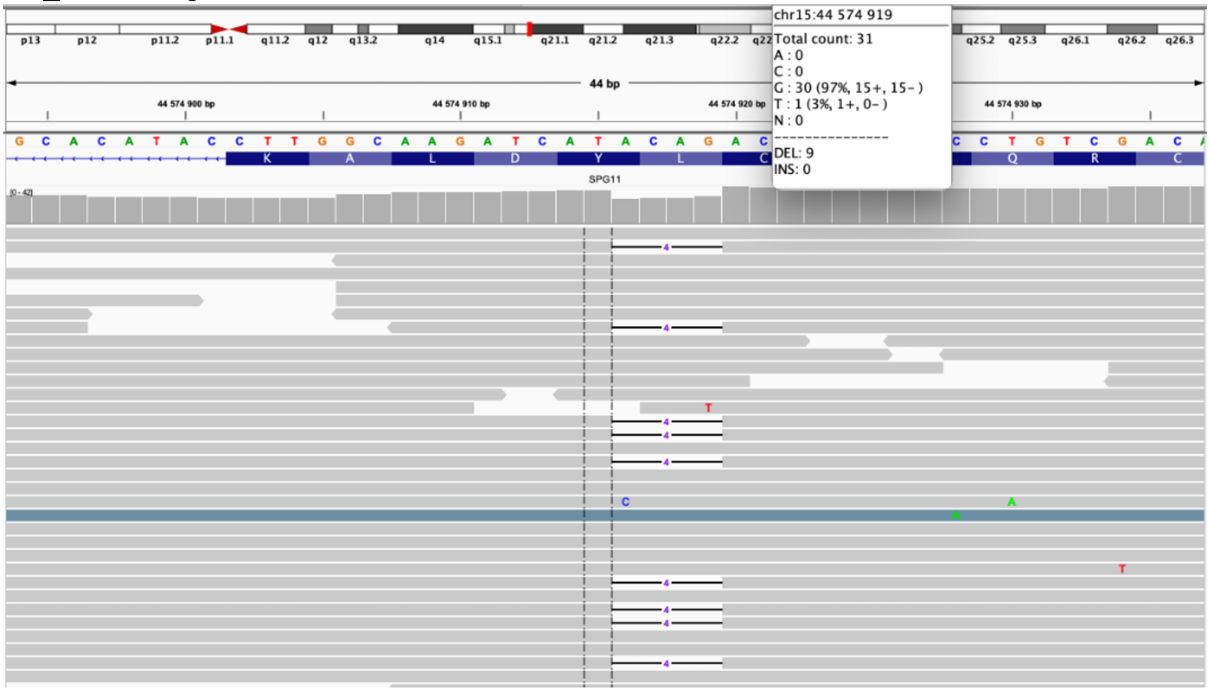

ii. ICGNMD_22 GRCh38 g.44613507dup (het) *SPG11* NP_079413.3:p.Glu1026ArgfsTer4

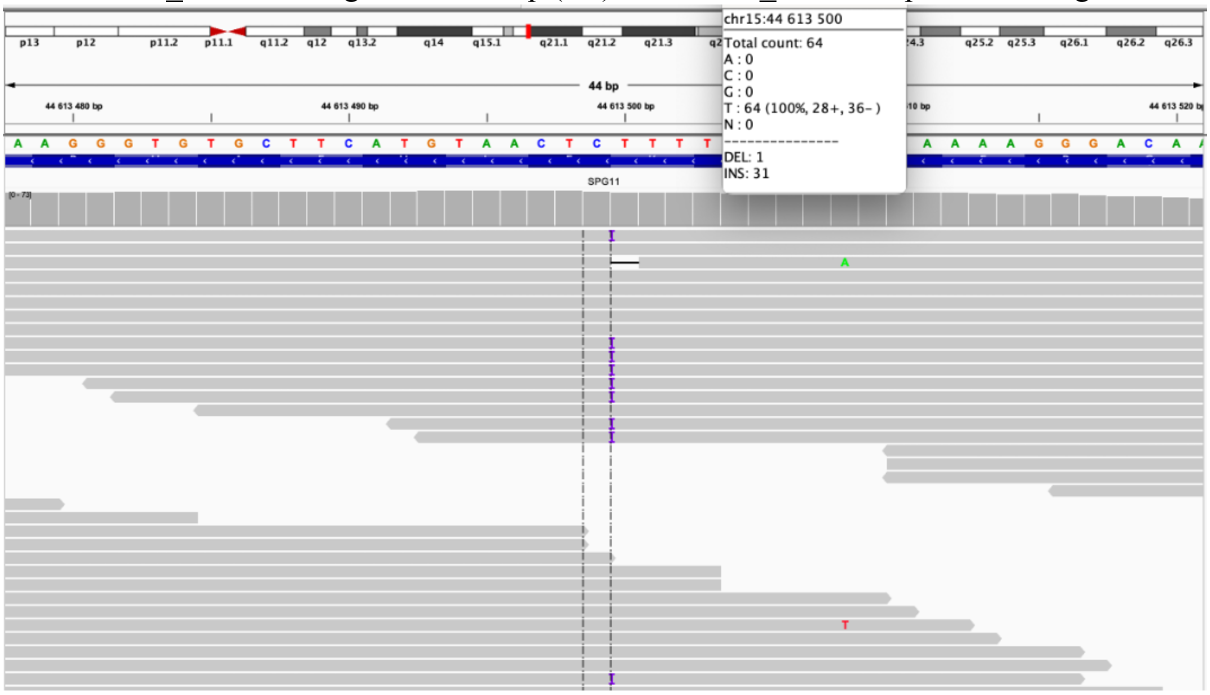

T. ICGNMD_26 GRCh38 g.44574917CAGA (hom) *SPG11* NP_079413.3:p.Leu1997MetfsTer60

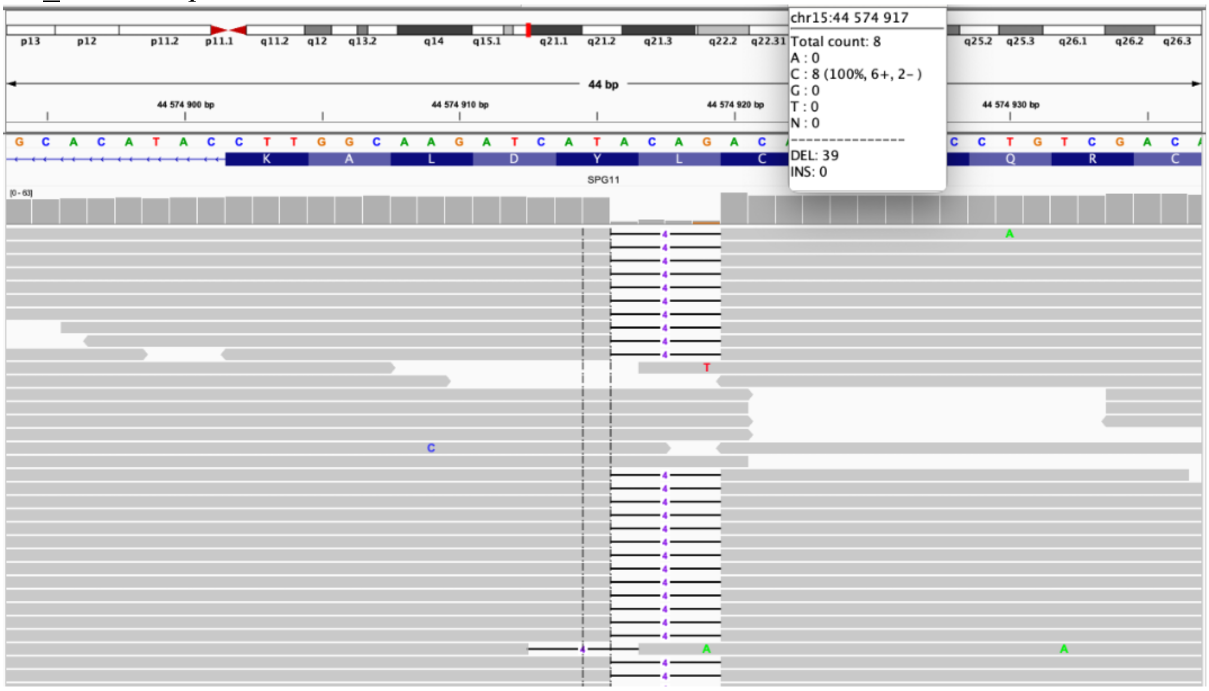

U. fam_009 GRCh38 g.44651634 A>G (hom) *SPG11* NP_079413.3:p.Leu438Pro

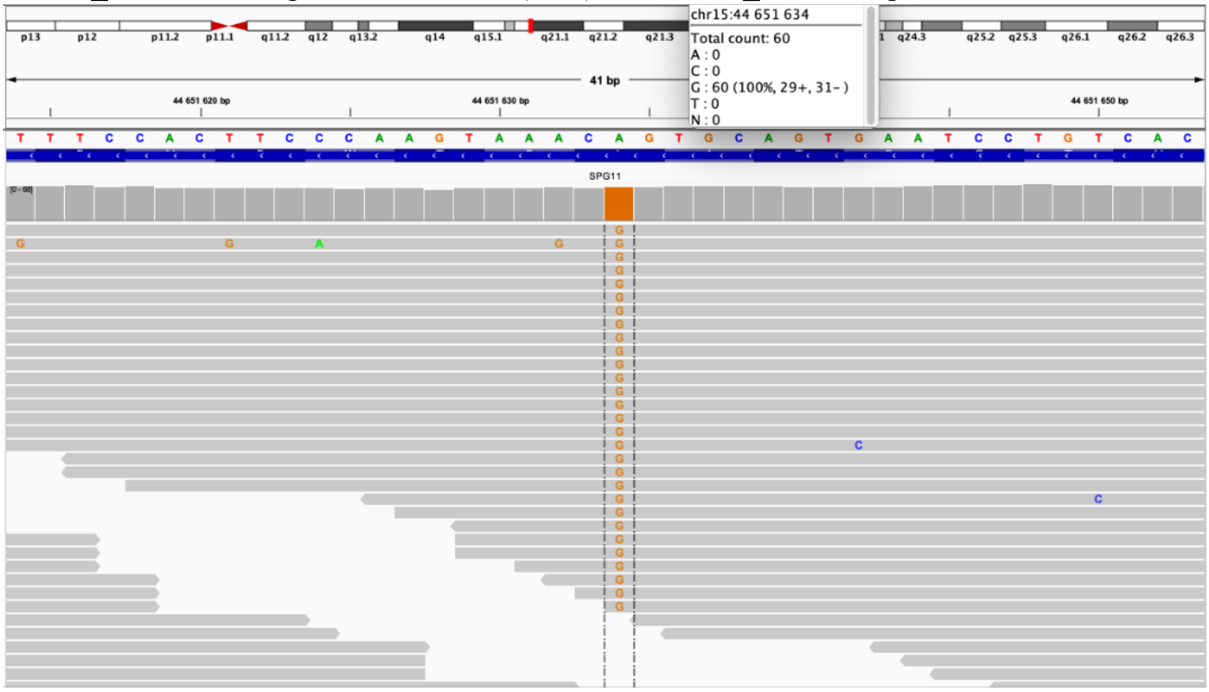

V. ICGNMD_8 GRCh382 g.53092049 C>T (hom) *RFT1* NP_443091.1:p.Gly494Ser

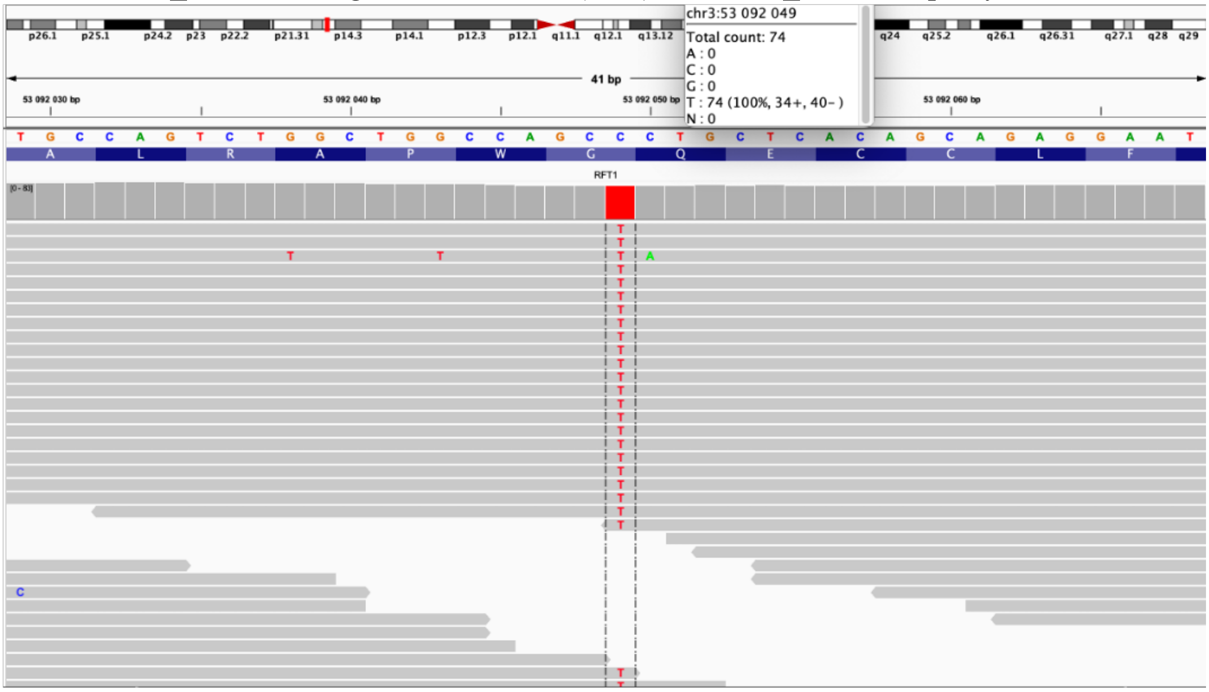

W. fam_195 GRCh38 g.95628493 C>G (hom) *ALDH18A1* NP_002851.2:p.Val451Met

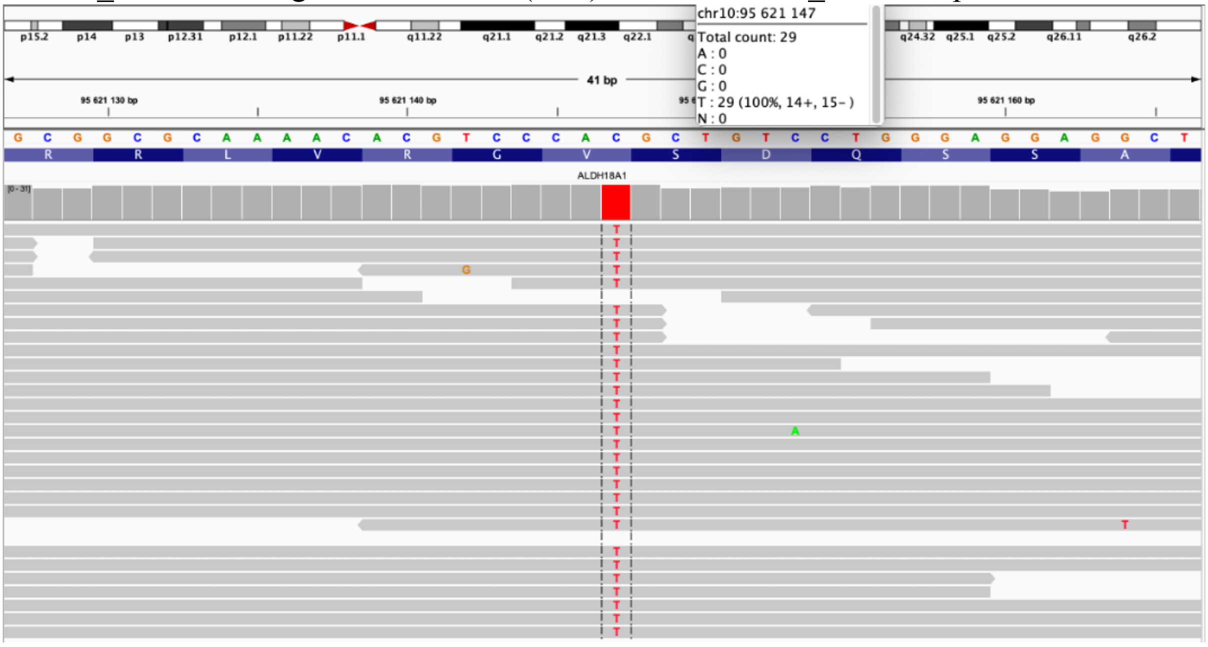

X. i. fam_122 GRCh38 g.64604713 T>C (het) *CYP7B1* p.His401Arg

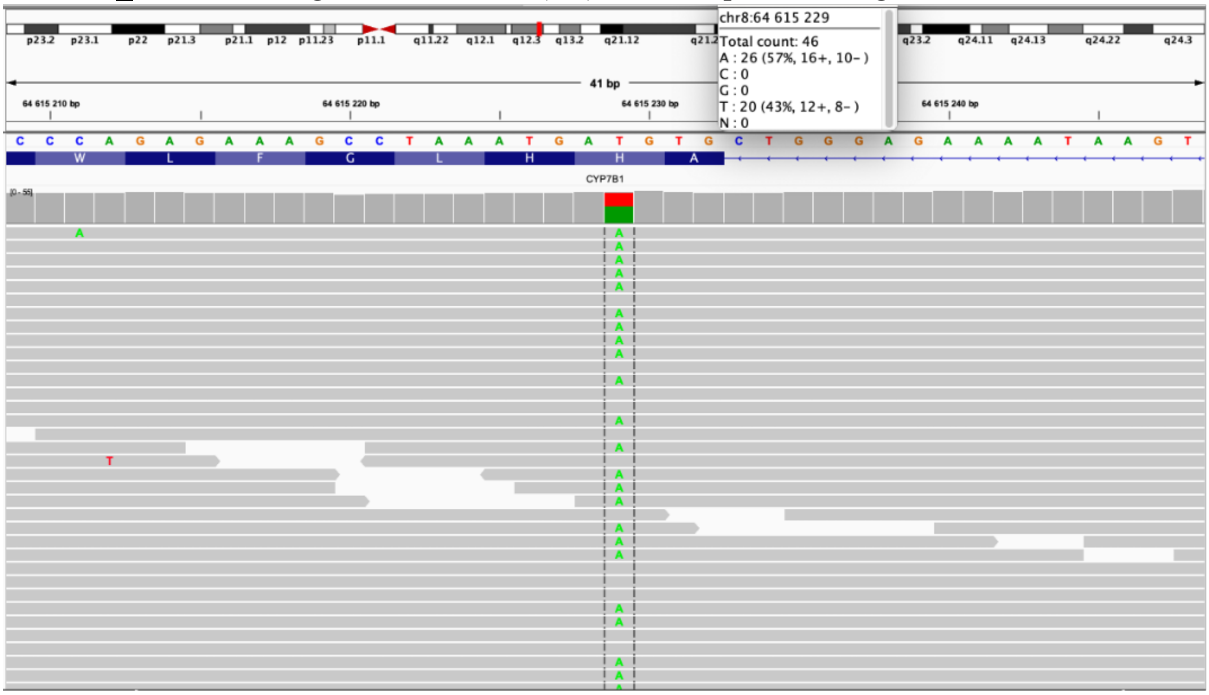

ii. fam_122 GRCh38 g.64615229 T >A (het) *CYP7B1* p.His285Leu

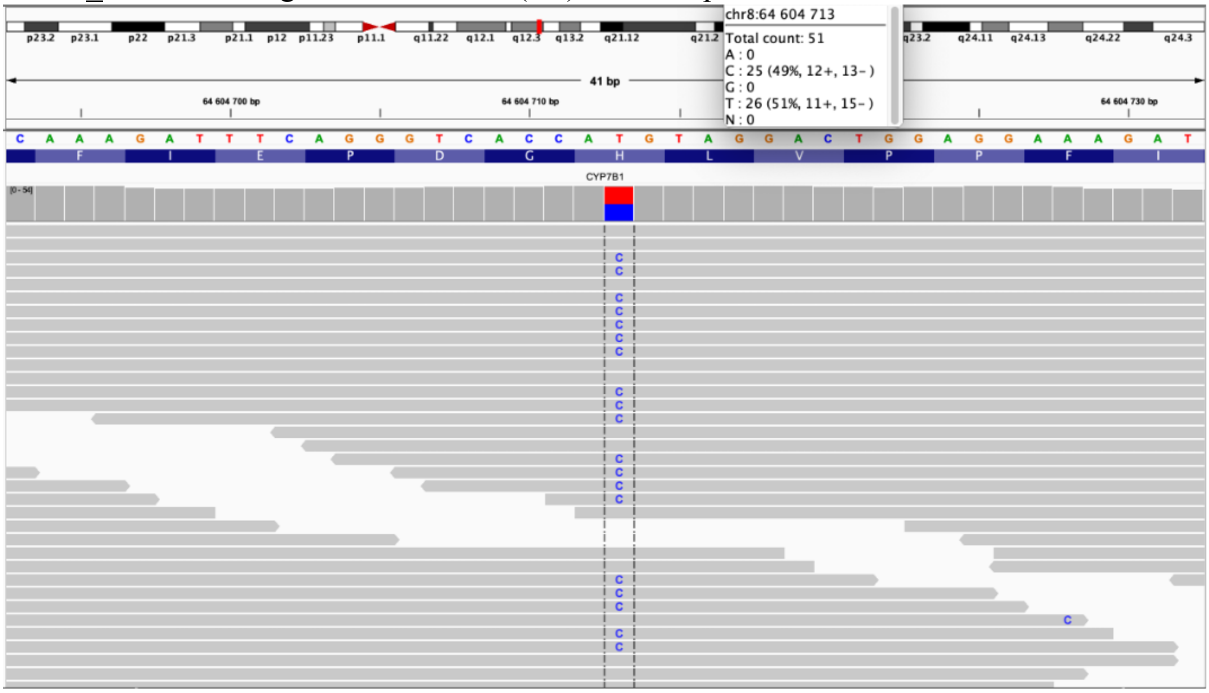

Y. fam_142 GRCh38 g.95628493 C>G (het) *ALDH18A1* NM_002860.4:c.809-1G>C

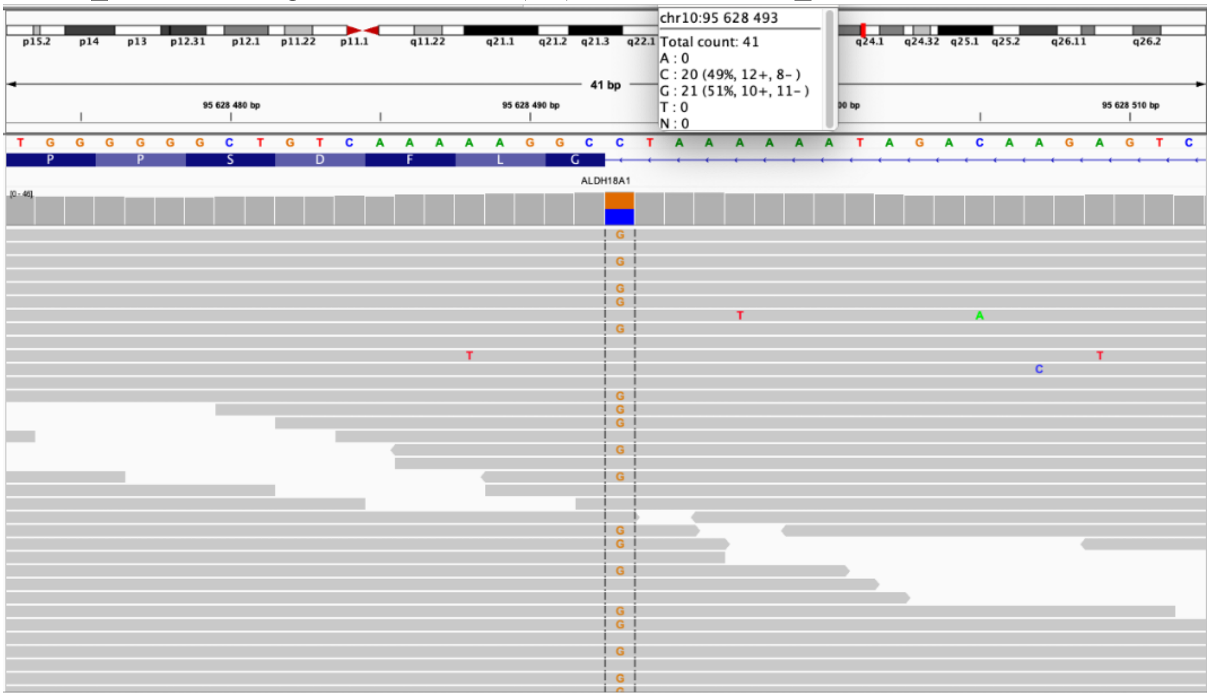

Supplement: Supplementary file 4 [file Image_1.pdf]
